# Growing functional artificial cytoskeletons in the viscoelastic confinement of DNA synthetic cells

**DOI:** 10.1038/s44286-025-00289-5

**Published:** 2025-10-07

**Authors:** Weixiang Chen, Siyu Song, Avik Samanta, Soumya Sethi, Christoph Drees, Michael Kappl, Hans-Jürgen Butt, Andreas Walther

**Affiliations:** 1 Life-Like Materials and Systems, Department of Chemistry, University of Mainz, Mainz, Germany; 2 Max Planck Institute for Polymer Research, Mainz, Germany; 3 Department of Chemistry, Indian Institute of Technology Kharagpur, Kharagpur, India

**Keywords:** DNA nanotechnology, Bioinspired materials, Self-assembly, Soft materials

## Abstract

Intracellular structures, such as cytoskeletons, form within a crowded cytoplasm with viscoelastic properties. While self-assembly in crowding is well studied, the effects of coupled viscoelastic environments remain elusive. Here we engineer all-DNA synthetic cells (SCs) with tunable viscoelastic interiors to investigate this phenomenon. We introduce facile DNA barcode engineering to selectively enrich DNA tiles with adjustable concentrations into SCs to form artificial cytoskeletons coupled to their interior. Distinct mechanistic differences in assembly occur compared with solution or simple crowding. Furthermore, we develop light, molecular and metabolic switches to direct structure formation and create self-sorted SC populations with distinct artificial cytoskeletons. These cytoskeletons strengthen SCs and support stable contacts with mammalian cells. By bridging molecular-scale DNA nanotube assembly with mesoscale condensate structures, our SCs provide a versatile platform to investigate self-assembly under viscoelastic confinement and to harness subcellular architectures for emerging applications.

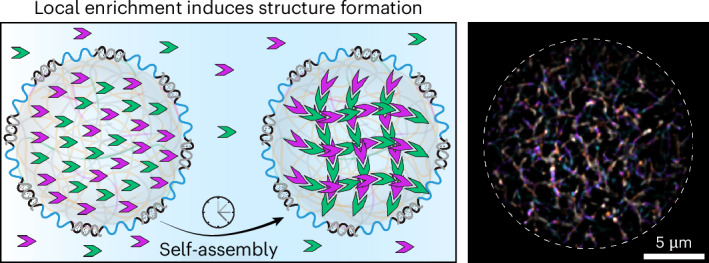

## Abstract (web summary)

Engineering structurally and functionally complex synthetic cells remains a key challenge. Here DNA condensate synthetic cells combine phase separation and DNA nanostructures to reveal how switchable artificial cytoskeletons assemble in viscoelastic confinements. These cytoskeletons improve the mechanical properties of synthetic cells and enable stable mechano-interfaces with mammalian cells.

## Main

Cells are compartmentalized dynamic systems that sustain out-of-equilibrium functions and structures by selective exchange of nutrients, signals and wastes with the environment^1–4^. The intracellular cytoplasm is a macromolecularly crowded interior with defined viscoelastic properties, which influences both catalysis and subcellular structure formation^5–8^. Fundamentally, viscoelasticity arises from the relaxation dynamics of macromolecules in solutions of different relaxation modes at different timescales, which is well described by Zimm, Rouse and Reptation models for macromolecular solutions at different concentration regimes^9^. The viscoelastic properties are in particular relevant to the formation of subcellular structures because the length and timescales of their formation may interface with the dynamics of the viscoelastic cytoplasm matrix^10–13^. These effects are beyond simple crowding. Mimicking defined crowded and/or viscoelastic environments in synthetic model systems, such as synthetic cells (SCs), may provide insights into cellular processes. Efforts have been dedicated to building SCs to tackle the challenge of mimicking cell-inspired structures and functions and to better understand cellular processes^14–19^. SCs can be obtained as water enclosed compartments from liposomes^20–23^, polymersomes^24,25^ or colloidosomes^26–28^ or from liquid–liquid phase separation and complex coacervation enriching polymers, proteins and nucleic acids^29–33^. The latter are suitable mimics of the intracellular environment, as they reproduce features of the cytoplasm regarding macromolecular crowding and viscoelastic properties^6,13,34^.

Construction of secondary structures inside SCs, such as cytoskeletons and membraneless organelles, is nontrivial due to the challenge of manipulating monomers and self-assembly inside the confinement^10,11,35–37^. Recent examples of building complex structures inside SCs include artificial cytoskeletons and droplet-like subcompartments^22,38–41^. In the context of this work, DNA nanotubes (DNTs)^42^ have been organized as an artificial cytoskeleton in water-in-oil emulsions^38^ and in liposomes^22,39,43^. Excluded volume effects by dextran or addition of Mg^2+^ have been used to induce bundling of DNTs^39,43^.

**Fig. 1:**
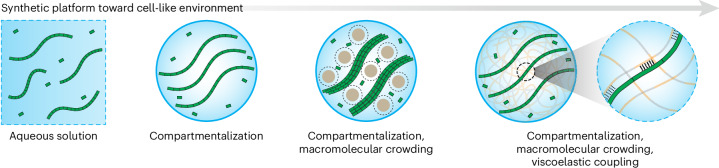
Self-assembly in distinct environments. The self-assembly of DNTs (shown as green tiles and fibers) was first studied in dilute aqueous solution or in compartments and then extended to crowded compartments (blue circles) containing macromolecular crowding agents (brown circles, and the dashed outlines indicate excluded volume). In this work, we introduce a crowded, viscoelastic compartment in which assembling DNT monomers are supramolecularly coupled to the surrounding viscoelastic matrix (polymer coil structure in background), enabling viscoelastic confinement.

The influence of macromolecular crowding on enzymatic catalysis and structure formation through excluded volume effects has been extensively studied^19,44–47^. Molecular crowding in fact requires absence of interactions between a colloidal species and the used crowder to form the excluded volume layer. The Asakura–Oosawa model is commonly used to describe depletion attraction dominated by an entropic gain^48,49^. However, in addition to being densely crowded, the cellular environment is also viscoelastic as a result of macromolecular relaxation processes. In contrast to the well-studied effects of crowding, much less is known about how viscoelasticity—superimposed on macromolecular crowding—affects self-assembly and structure formation (Fig. 1). In this case, coupling may occur purely through physical constraints, akin to soft interactions, or in regimes with strong coupling, for instance, by tethering the building blocks to a matrix. We focus on the second aspect because bonding of the self-assembling DNT tiles is essential for their enrichment in SCs and studying their assembly. Only a few recent studies have started to look at structure formation in macroscopic gels, hence in situations where soft coupling can be studied without the need for enriching building blocks as in case of SCs^50–53^. Bringing viscoelastically coupled self-assembly approaches to the field of SCs requires developing SC models that allow manipulation of their viscoelastic properties and controlled enrichment of building blocks, which will benefit both the fundamental understanding and the improvement of the engineering principles of SCs toward structural and functional properties.

Here, we develop a crowded all-DNA SC system to study the formation of artificial cytoskeletons in viscoelastic confinement under strong coupling conditions, by combining DNA nanotechnology-based DNA self-assembly at the molecular scale with DNA polymer phase separation at the mesoscale. The SC is characterized as a confined, semi-dilute polymer solution, whose viscoelasticity is intrinsically related to concentration, temperature and additional crosslinking. The SCs allow selective enrichment of cytoskeleton monomers that are bonded to the internal SC matrix using short DNA barcode domains. The SCs are formed by a temperature-induced condensation process and contain a liquid interior composed of long single-stranded DNA (ssDNA) stabilized by a permeable DNA hydrogel shell^17,41,54,55^. We will first introduce a co-phase-separation approach to integrate multiple barcodes with controlled concentrations into the all-DNA SCs. We then demonstrate how artificial cytoskeletons grow in these confinements and how the concentration of DNA tiles and the viscoelastic properties regulate the assembly process. We further showcase the versatility of this approach to control the selective enrichment of different cytoskeletal building blocks to achieve self-sorted cytoskeletons within SCs and self-sorted SCs containing distinct artificial cytoskeletons in SC communities. We demonstrate the functional benefit of forming artificial cytoskeletons to enhance the stiffness of SCs, as well as providing stability during SC–mammalian cell contact when building a mechano-interface to cells using arginine–glycine–aspartic acid (RGD) peptide–integrin binding. Our molecular engineering approach provides versatile SC systems to fundamentally study self-assembly in viscoelastic confinement and guide the design of future SCs with emergent functions via embedded secondary structures.

## Results

### Barcode engineering inside SCs by co-phase separation

The preparation of all-DNA core–shell SCs builds on our original finding of the lower critical solution temperature behavior of adenine-rich ssDNA polymers^54,55^. Previously, we had shown that heating a mixture of adenine-rich and thymine-rich ssDNA polymers, that is, p(A_20_-m)_*n*_ and p(T_20_-k)_*n*_, induces condensation of p(A_20_-m)_*n*_ into spherical condensates. During cooling the p(T_20_-k)_*n*_ hybridizes on the periphery of the p(A_20_-m)_*n*_ droplets, trapping the core–shell SCs with homogenous distribution of p(A_20_-m)_*n*_ in the core and p(T_20_-k)_*n*_ at the shell^19,54^. A_20_ and T_20_ are repeats of 20 nucleotides of adenine and thymine, whereas ‘m’ and ‘k’ are short ssDNA barcodes embedded in the repeating unit of the polymer chains. The subscript *n* denotes the number of repeats, which is around 70 for adenine-rich DNA polymers and above 80 for thymine-rich DNA polymers (Supplementary Fig. 1). The barcodes allow selective functionalization of the core and shell by complementary ssDNA.

**Fig. 2:**
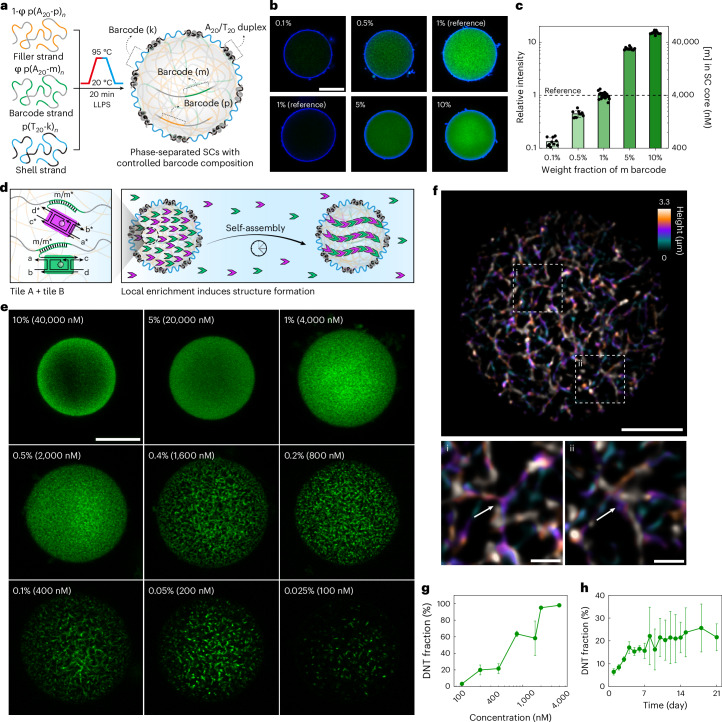
Barcode engineering of core–shell DNA SCs for controlled enrichment and cytoskeleton formation. **a**, A schematic of the co-phase separation approach for preparing SCs containing two core barcodes (m and p). LLPS, liquid–liquid phase separation. **b**, Representative CLSM images of SCs with varying weight fractions of m barcode (0.1–10%), labeled by Atto488-m* (green, core) and Atto647-k* (blue, shell). Note that laser settings are identical for each row of the images, enabling direct comparison of internal fluorescence intensity and quantification of relative m barcode concentration. **c**, A quantification of internal m barcode concentration ([m]) as a function of p(A_20_-m)_*n*_ weight fraction in mixture with p(A_20_-p)_*n*_ (mean ± s.d., sample size, *n* = 10 SCs measured). **d**, A schematic of barcode-mediated enrichment of DNA tiles and cytoskeleton formation. **e**, Representative CLSM images showing distinct cytoskeleton morphologies at different [m]. **f**, A SIM image of artificial cytoskeletons formed in an SC ([m] = 400 nM). The color-coded *z*-projection image reveals branching as indicated by white arrows in the zoomed-in images (i and ii). **g**, Increasing DNT fraction in SCs with higher [m] after 20 ± 1 days of growth (mean ± s.d., *n* = 4–10 SCs measured at each concentration). **h**, A quantification of DNT growth at [m] = 400 nM. The DNT fraction is quantified by the fraction of pixel counts inside SCs over a threshold intensity (mean ± s.d., *n* = 6–14 SCs measured at each day). Experiment temperature, 20 °C. The DNTs have two colocalized fluorescent labels (Atto488 and Atto647). Only Atto488 (green) is shown for highest resolution and better contrast. Scale bars, 10 μm for **b** and **e**, 5 μm for **f** and 2 μm for **f**(i,ii) in zoomed-in SIM. Source data

The concentration of the m barcode ([m]) in the core is a fixed value determined by the thermodynamics of phase separation of p(A_20_-m)_*n*_. As we show below, tuning the barcode concentration is required to precisely control the enrichment of building blocks. Hence, to finetune [m] inside the core matrix of an SC, we introduce a new co-phase-separation method by mixing various adenine-rich ssDNA polymers with different barcode sequences. To this end, we synthesized two additional adenine-rich DNA polymers, namely p(A_20_-p)_*n*_ and p(A_20_-s)_*n*_ (Supplementary Fig. 1 and Supplementary Method 1). We first focus on binary mixtures of p(A_20_-p)_*n*_ and p(A_20_-m)_*n*_ in different ratios (Fig. 2a). As the thermodynamic driving force for phase separation is encoded in the A_20_ repeats present in both ssDNA polymers, we expected similar temperature-dependent condensation of both p(A_20_-p)_*n*_ and p(A_20_-m)_*n*_, as well as co-condensation of them due to their chemical similarity.

To verify our concept, we prepared a series of SCs with different ratios of p(A_20_-m)_*n*_ and p(A_20_-p)_*n*_, while keeping their total concentration constant at 0.5 g l^−1^. p(A_20_-p)_*n*_ is introduced as a nonfunctional filler strand, which constitutes the majority weight fraction (from 90% to 99.9%), while p(A_20_-m)_*n*_ ranges from 10% to 0.1%. We labeled these SCs with Atto488-m* and Atto647-k* for microscopy and found morphologically similar SCs for all mixtures, independent of the used ratio of p(A_20_-m)_*n*_/p(A_20_-p)_*n*_ (Fig. 2b). Quantification of the Atto488 fluorescence in the core shows a clear dependence of the fluorescence intensity on the weight fraction of p(A_20_-m)_*n*_ used (Fig. 2b,c). The intensity differences span three orders of magnitude, confirming that the [m] can be controlled by simply adjusting the ratio of p(A_20_-m)_*n*_/p(A_20_-p)_*n*_. Mesoscopic demixing of p(A_20_-m)_*n*_ and p(A_20_-p)_*n*_ is absent. Based on titration measurements, the absolute [m] inside the SC core ranges from 100 nM for 0.025% of p(A_20_-m)_*n*_ to 40,000 nM for 10% of p(A_20_-m)_*n*_ (Supplementary Fig. 2 and Supplementary Note 1). The total concentration of all A_20_-*x* (where *x* is m or p) repeating units inside SCs is around 400 μM (5 g l^−1^)^56^ (Supplementary Note 1).

### Formation of artificial cytoskeletons inside SCs

We use DNTs formed by DNA tiles as cytoskeleton-mimic. Such DNTs are versatile and robust systems, and their growth has been demonstrated in solution as well as in aqueous compartments^38,39,42^. For the integration into all-DNA SCs, we first focus on identifying the correct concentration, and establishing conditions providing enrichment inside the SCs. We start by using a binary DNT tile system^38,42^ composed of tile A and tile B (Supplementary Fig. 3), which are mutually complementary but not self-complementary based on their sticky end interaction. For visualization and enrichment inside the SCs, we modified both tiles with fluorescent labels (Atto488 for tile A and Atto647 for tile B) and a dangling strand complementary to the m barcode inside the SCs (Fig. 2d). The two DNA tiles were annealed separately so that no fibrils can form during annealing.

We then mixed individual SCs having different [m] in the core with 20 nM DNA tiles to enrich them inside the SCs and avoid undesired DNT formation in solution. Depending on the SCs used (different [m]), the DNA tiles can be over-stoichiometric, stoichiometric, or substoichiometric with respect to the m barcode. A sufficient concentration of added DNA tiles is important to facilitate their enrichment inside SCs, particularly at low total [m] (Supplementary Fig. 4). Supplementary Table 1 lists details of the experimental settings. As expected, the tiles do not form any observable structure in solution due to their low concentration. Rather, they accumulate in SCs via m/m* recognition to controlled concentrations programmed by the m content of the co-phase-segregated p(A_20_-m)_*n*_ and p(A_20_-p)_*n*_. This selective enrichment of the tile concentration is of critical importance, as it enables structure formation exclusively within the compartment. By contrast, when mixing SCs with DNA tiles without barcodes, a preferred partitioning of the tiles into the SCs does not occur (Supplementary Fig. 5). This underscores that chemical interactions are needed to sequester the DNT tiles into the SCs, allowing to study structure formation under strong coupling conditions to the matrix.

**Extended Data Fig. 1:**
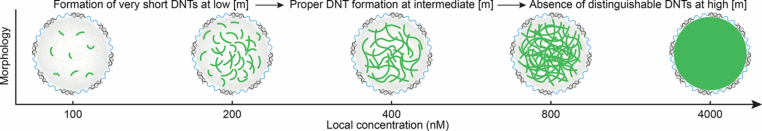
Phase diagram of cytoskeleton morphologies inside SCs as a function of [m]. At low concentrations (<200 nM), DNA tiles assemble only into short fibers. When the local concentration increases to ~400 nM, DNTs form well-defined network structures inside SCs. At higher concentrations (>800 nM), individual DNTs become indistinguishable. The absence of DNT formation at high concentration is due to the excessive tile concentration and the freezing of dynamics required for rearrangement in the SC interior.

We compare DNT growth in the SCs after 20 ± 1 days under a confocal laser scanning microscopy (CLSM) (Fig. 2e,g). Three main regimes can be delineated (Extended Data Fig. 1): (1) absence of distinguishable DNTs at high [m], (2) proper DNT formation at intermediate [m] and (3) formation of very short DNTs at low [m]. In SCs with 10% m barcode ([m] = 40,000 nM), the tiles cannot fully address all barcodes due to their substoichiometric amount (50%) (Supplementary Table 1). Therefore, a higher concentration at the rim of the SCs can be observed (Fig. 2e). DNT formation is absent even after 20 days of incubation. Down to SCs containing 0.5% m barcode ([m] = 2,000 nM), no clear fibrillar structures are formed. We suggest that the absence of fiber formation in this first regime is due to the excessive concentration and the freezing of dynamics required for rearrangement in the viscoelastic interior. The sticky end interactions of the tiles prevent reorientation to form well-defined structures. Increasingly arrested dynamics of the SC interior at higher [m] can be confirmed by fluorescence recovery after photobleaching (FRAP) experiments (Supplementary Fig. 6b–d). Upon further lowering of [m] in the SCs to 0.4% (1,600 nM), clear fibrils start to appear in a very dense state. When [m] inside the SCs reaches 0.1% (400 nM), the formed artificial cytoskeleton becomes clearly discernible in CLSM. A further reduction of [m] leads to insufficient concentrations of the DNA tiles, which ultimately limits the growth of any artificial cytoskeleton to short fibers. This underscores the importance of precisely engineering the enrichment and local concentration of building blocks to ensure proper structure formation in crowded and viscoelastic environments. Details of the process also depend on the temperature of assembly. For instance, higher temperatures facilitate the formation of DNTs even at higher loading concentrations ([m] = 10%) on account of higher dynamics (Supplementary Fig. 7).

**Extended Data Fig. 2:**
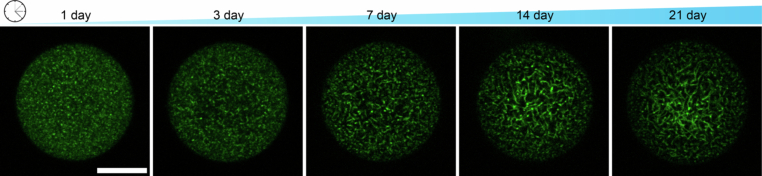
Growth kinetics of artificial cytoskeletons inside SCs. Time series CLSM images for the growth of artificial cytoskeletons inside an SC with [m] = 0.1% (400 nM) and [p] = 99.9% (400,000 nM). Experiment temperature, 20 °C. Scale bar, 10 μm.

Superresolution imaging by structured illumination microscopy (SIM) provides further details and reveals a substantially higher degree of branching of the DNTs in SCs compared with DNTs formed in aqueous solution (Fig. 2f, *z*-section, and Supplementary Fig. 3b). A time-series CLSM reveals massive nucleation events at an early assembly stage, forming bright nuclei, which slowly evolve to fibrils after 2–3 weeks (Fig. 2h, Extended Data Fig. 2 and Supplementary Fig. 8a). This observation suggests that fibril growth occurs by end-to-end joining and rearrangement of short DNT segments and not by classical addition of DNT tiles to seeds and growing DNTs in solution^57–59^. This is because nucleation into short segments has plenty of time to occur in small viscoelastic volumes, whereas the subsequent slow growth requires relaxation of the polymeric and viscoelastic ssDNA matrix and larger scale rearrangement to accommodate structure formation. The growth of DNTs is dominated by the tiles bonded via m/m* hybridization inside SCs, whereas free tiles in solution only have a minor contribution (Supplementary Fig. 9). The continuous structural evolution of the artificial cytoskeletons over time confirms that certain dynamics exist in the system. This is also verified by a 2-day long FRAP experiment that shows recovery of the cytoskeleton (Supplementary Fig. 6a). Crosslinking the entire SC matrix via the p barcodes of co-assembled p(A_20_-p)_*n*_ prevents assembly, as the SC matrix transforms from a semi-dilute polymer solution to a hydrogel network, and the SC’s viscoelastic properties are largely altered from more viscous to more elastic (Supplementary Fig. 10). Importantly, clustering of DNTs into bundles is not observed as has been reported for simple crowders (dextran)^39^. The absence of bundling is due to the fact that the tiles are all bonded to the p(A_20_-m)_*n*_ core strands to prevent segregation (Fig. 2d). By contrast, when nonbinding DNA tiles assemble in a concentrated p(A_20_-p)_*n*_ solution at 5 g l^−1^, which nominally represents the SC interior, bundling of DNTs occurs. This is similar to the aforementioned simple crowders such as PEG or dextran^39^ (Supplementary Fig. 11).

The SCs can be considered a semi-dilute polymer solution above the overlap concentration (c*) (5 g l^−1^, average molecular weight around 900 kDa). Depending on whether taking the end-to-end distance or the radius of gyration as basis, the overlap concentration can be calculated to c*_lower_ ≈ 0.28 g l^−1^ and c*_upper_ ≈ 4.43 g l^−1^, respectively (Supplementary Note 2). The viscoelastic properties can thus be described by established relationships for semi-dilute polymer solution. For instance, zero shear viscosity increases with concentration as well as decreases with higher temperature on account of the Arrhenius relationship. On a molecular level, this relates to polymer relaxation dynamics. Since matrix rearrangement is critical to accommodate growing DNTs, we hypothesized an impact of viscosity on DNT growth dynamics. We note that for noncrosslinked SCs, the assembly process is so slow that it couples to the low-frequency regime that probes rather the viscous properties of the SC matrix.

To understand the impact of viscosity at constant temperature, we designed SCs composed of three pA strands (5 g l^−1^) and degraded one of them using selective enzyme degradation (Supplementary Fig. 12). This yields SCs with only 3 g l^−1^ pA in the core, while [m] is the same as for the standard SCs. Afterward, we incubated DNA tiles with SCs before (5 g l^−1^) and after degradation (3 g l^−1^). FRAP experiments confirm enhanced dynamics for SCs with 3 g l^−1^ as seen by ~40% increase in the reciprocal of half recovery time (1/*t*_1/2_), thus confirming the impact of reduced concentration on dynamics (Supplementary Fig. 12f,g). This leads to a marked increase in the DNT growth kinetics inside the SCs with 3 g l^−1^, wherein DNTs are clearly visible within 3 days of assembly time (Supplementary Fig. 12e,h,i)—approximately half the time required for standard SCs (7 days). This underscores the importance of matrix dynamics on the self-assembly timescale.

Temperature has a further influence on semi-dilute polymer solution dynamics and therefore impacts the corresponding viscoelasticity. We studied DNT growth in standard SCs with 5 g l^−1^ at 20 °C, 25 °C, 30 °C and 35 °C. In general, temperature has two effects: first, higher temperature enhances reptation dynamics and thereby contributes to faster stress relaxation and lower viscosity, as can be probed by FRAP (Supplementary Fig. 13e,f). Second, higher temperature dynamizes DNA duplexes and lowers the equilibrium fraction of binding according to the melting temperature (*T*_m_). Profound differences in the growth kinetics and final DNT density inside SCs occur (Supplementary Fig. 13a–c). Temperature increase accelerates the DNT growth when going from 20 °C to 25 °C and 30 °C; however, DNT formation is fully absent at 35 °C (Supplementary Fig. 13d). For instance, at day 3, DNTs are barely visible at 20 °C, whereas they have clearly formed already at 25 °C and 30 °C. Moreover, the DNT density inside SCs starts to decrease at 30 °C. This can be explained as follows: faster growth originates from lower viscosity at higher temperatures. However, once reaching 30 °C, increased reaction kinetics due to fast reptation are outbalanced by the weaker sticky end interactions between the DNT tiles responsible for their assembly (interaction only based on 5 base pairs (bp)) and furthermore by the lower DNT tile recruitment due to insufficient m/m* interactions to sequester DNTs into the SCs. At 35 °C, the weakened DNA interactions dominate the system and prevent assembly. Therefore, we determine 25 °C to be the optimum temperature, balancing quickest growth and high density of DNTs inside SCs.

Interestingly, the dynamic properties of the final DNT cytoskeleton inside SCs are different at different temperatures (Supplementary Video 1). When placing DNT-containing SCs grown at 25 °C to 20 °C and 30 °C, the DNT structure is very stable and shows almost no movements over 1 h at 20 °C, whereas considerable dynamics are visible at 30 °C. This enhanced movement relates to more dynamic interactions at the m/m* interface by which the cytoskeleton is bonded to the SC matrix.

### Stimuli-induced and metabolic cytoskeleton formation in SCs

**Fig. 3:**
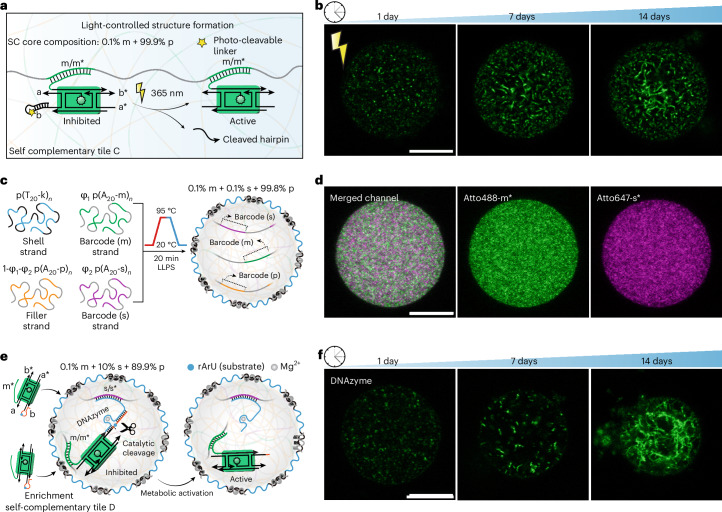
Light-triggered and DNAzyme-catalyzed metabolic formation of artificial cytoskeletons inside SCs. **a**, The design of tile C for light-controlled growth of artificial cytoskeletons. **b**, Time-series CLSM images showing cytoskeleton formation inside an SC ([m] = 400 nM) upon UV illumination. **c**, A schematic of the preparation of SCs containing three core barcodes (m, p and s). **d**, Representative CLSM images showing an SC containing 99.8% p (400,000 nM), 0.1% m (400 nM) and 0.1% s (400 nM) barcodes, addressed by Atto488-m* and Atto647-s*. **e**, The design of tile D for DNAzyme-catalyzed metabolic formation of artificial cytoskeletons in SCs. **f**, Time-series CLSM images showing cytoskeleton formation inside an SC ([m] = 400 nM) catalyzed by DNAzyme. Experiment temperature, 20 °C. Scale bars, 10 μm for **b**, **d** and **f**.

Next, to achieve controlled formation of artificial cytoskeletons inside SCs, we engineer control mechanisms to the DNT system, notably light and strand displacement triggers, as well as a metabolic induction. For that, we move to DNTs formed solely by one tile, and first designed a light-activated tile C (Supplementary Fig. 14). Tile C has sticky ends that are self-complementary, inhibited by a hairpin at one of its sticky ends (Fig. 3a) that contains a photo-cleavable linker (PCL) in its loop. The design utilizes an important principle of DNA duplex stability: a double-stranded DNA (dsDNA) in a hairpin structure is thermally more stable than the same dsDNA formed by two ssDNAs. We designed the hairpin to have 5 bp in its stem region with a *T*_m_ around 60 °C. The same dsDNA formed by corresponding ssDNA is however unstable even down to 0 °C (Supplementary Fig. 14). Hence, without ultraviolet (UV) illumination, the hairpin is stable and inhibits the sticky end interactions. Once the DNA tiles are exposed to UV light, the PCL is cleaved, and the resulting 5 bp dsDNA becomes thermally unstable and melts. This activates the DNA tiles for self-assembly into fibrils. To realize light-activated formation of artificial cytoskeletons inside the SCs, we charged SCs with 10 nM tile C, followed by illumination at 365 nm. As expected, artificial cytoskeletons gradually form over time (Fig. 3b and Supplementary Fig. 8c). By contrast, a control without illumination only shows small aggregates after 14 days, probably caused by ambient light pollution over long experimental time (Supplementary Fig. 15).

For integration of multiple structural units and metabolic functions within a single SC, we considered to generalize our co-phase separation approach to a ternary system. For that, we designed and synthesized an additional adenine-rich ssDNA polymer with a new barcode, namely, p(A_20_-s)_*n*_ (Supplementary Fig. 1). Applying the same co-phase-separation strategy, we mixed p(A_20_-p)_*n*_, p(A_20_-m)_*n*_, p(A_20_-s)_*n*_ and p(T_20_-k)_*n*_ together and prepared SCs via a temperature ramp (Fig. 3c). The majority of the SC core is p(A_20_-p)_*n*_ (99.8%), whereas 0.1% p(A_20_-m)_*n*_ and 0.1% p(A_20_-s)_*n*_ are homogeneously distributed at 400 nM each. p(T_20_-k)_*n*_ stabilizes the shell. CLSM confirms the successful encapsulation and homogenous distribution of both m and s barcodes inside a single SC (Fig. 3d).

We then designed a metabolic SC that can achieve metabolic structure formation by embedding a DNAzyme^60,61^ via the s barcodes. The DNAzyme is engineered to recognize the hairpin of a protected DNA tile D containing two RNA point mutations (rArU) (Fig. 3e and Supplementary Fig. 16). Tile D has an affinity via the orthogonal m barcodes. Indeed, once the DNAzyme and tile D meet in the same SC, the DNAzyme cleaves the hairpin at the RNA mutation and enables artificial cytoskeleton formation inside SCs by melting off the protective hairpin part (Fig. 3f and Supplementary Fig. 8d).

**Fig. 4:**
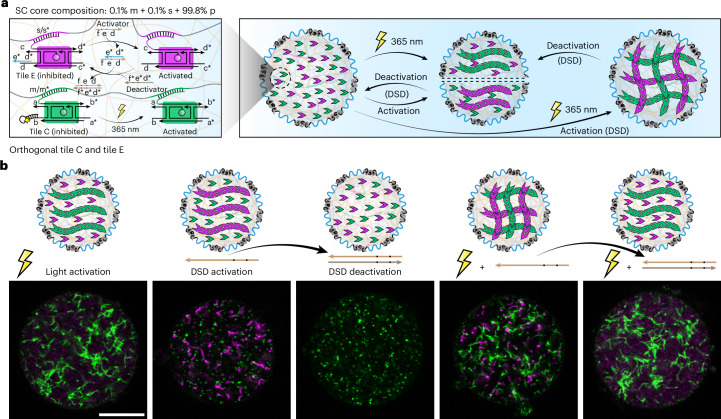
Dual cytoskeleton formation in SCs via multiple barcodes and orthogonal stimuli. **a**, A schematic of tile C and tile E enrichment by distinct barcodes (m and s), enabling orthogonal triggering of cytoskeleton assembly and disassembly. **b**, Schemes and representative CLSM images showing light- and DSD-triggered formation and deconstruction of cytoskeletons in SCs containing 400 nM m and 400 nM s barcodes. The green channel is for Atto488 (tile C), and the magenta channel is for Atto647 (tile E). Experiment temperature, 20 °C. Scale bar, 10 μm for **b**.

The ternary barcode SC system also enables the assembly of orthogonal cytoskeleton mimics within the same entity, similar as to how real cells can organize distinct fibrillar structures. As a proof of concept, we introduce a second one-tile DNT system based on tile E with a dangling strand that binds to the s barcode (Supplementary Fig. 17). This tile is inhibited by an inhibitor strand at one of its sticky ends and can be activated by an activator strand via a DNA strand displacement (DSD) reaction to initiate artificial cytoskeleton growth (Fig. 4a). After charging the SCs with both the light-activatable tile C and the DSD-switchable tile E, orthogonal triggers selectively control the formation of two different artificial cytoskeletons in the same compartment (Fig. 4b and Supplementary Fig. 8b). As expected, the sole formation of green cytoskeletons by tile C is visible after applying light stimulation. By contrast, only magenta cytoskeletons form when the appropriate activator strand is provided for the DSD reaction. Furthermore, the DSD-activated cytoskeletons can even be deconstructed by adding a deactivator strand, which displaces the activator strand and sets free the inhibitor strand to bind to the sticky ends for disassembly of the whole structure. Applying both stimuli at the same time results in formation of two orthogonal artificial cytoskeletons in the same SCs.

### Programmable selectivity in diverse SC communities

**Fig. 5:**
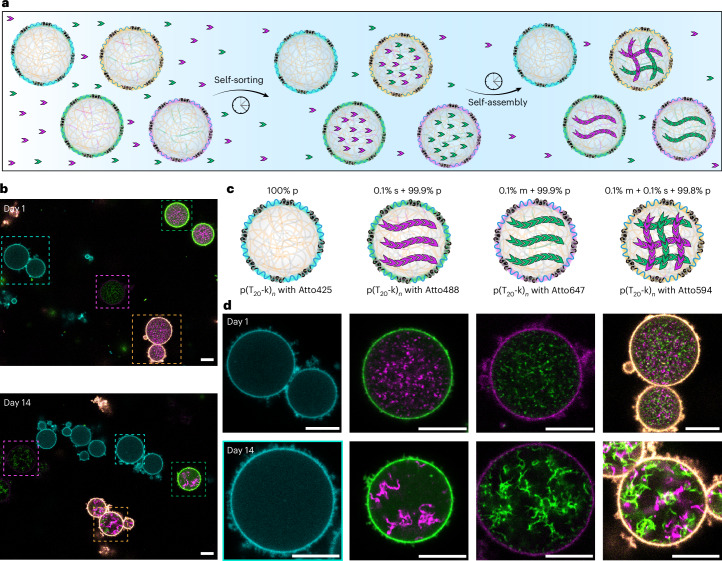
Selective uptake and growth of artificial cytoskeletons in differentiated SCs from a species pool of cytoskeletal monomers. **a**, A schematic of DNA tiles self-sorting into SCs from a common pool via programmed molecular recognition, followed by compartment-specific assembly. **b**, Overview CLSM images showing selective uptake and cytoskeleton growth (tile C and tile E) in SCs with different barcodes, distinguished by distinct shell labels. **c**, Schematics of SCs with different barcodes and shells labeled by p(T_20_-k)_*n*_ carrying different in-chain fluorophores. **d**, Corresponding zoomed-in CLSM images of SCs in the dashed boxes in **b** on day 1 and day 14. CLSM channels: Atto425 (cyan), Atto488 (green), Atto594 (yellow) and Atto647 (magenta). Experiment temperature, 20 °C. Scale bars, 10 μm for **b** and **d**.

Finally, we hypothesized that the precise selectivity for enrichment in our SCs can be exploited on a system level to generate different populations of cytoskeleton-mimic-containing SCs in the same system by selective enrichment in a pool of different DNT building blocks. To this end, we first synthesized a series of p(T_20_-k)_*n*_ with different in-chain fluorophores to label the SC shells (Supplementary Fig. 1). Next we prepared four different SCs, where the differently labeled p(T_20_-k)_*n*_ shells serve as identifiers for SCs containing different barcodes in the core for enrichment of different DNT systems: (1) cyan-shell SCs without any addressable barcode to enrich DNT tiles as control, (2) green-shell SCs with the s barcode to enrich tile E for DSD activation, (3) magenta-shell SCs with the m barcode to enrich tile C with the PCL and (4) yellow-shell SCs with the m and s barcodes together. Subsequently, we mixed all different SCs and added tile C and tile E in the same solution (Fig. 5a–d). Indeed, the SCs accumulate their corresponding tiles selectively, and, after triggering with the appropriate stimuli, self-sorted populations occur. The cyan-shell SCs containing only the p barcode do not show enrichment of any tiles and thus no cytoskeleton. The SCs with s or m barcode enrich their corresponding tiles E or tile C, respectively, which grow into artificial cytoskeletons in the distinct SCs over time. The SCs with both m and s barcode enrich both tiles and form two different kinds of cytoskeletons inside the same compartment. This demonstrates ultimately the strength of the co-phase-separation approach for barcoding individual populations and for reaching selectivity in a diverse community of SCs.

### Mechanical properties of SCs and durable SC-cell contacts

**Fig. 6:**
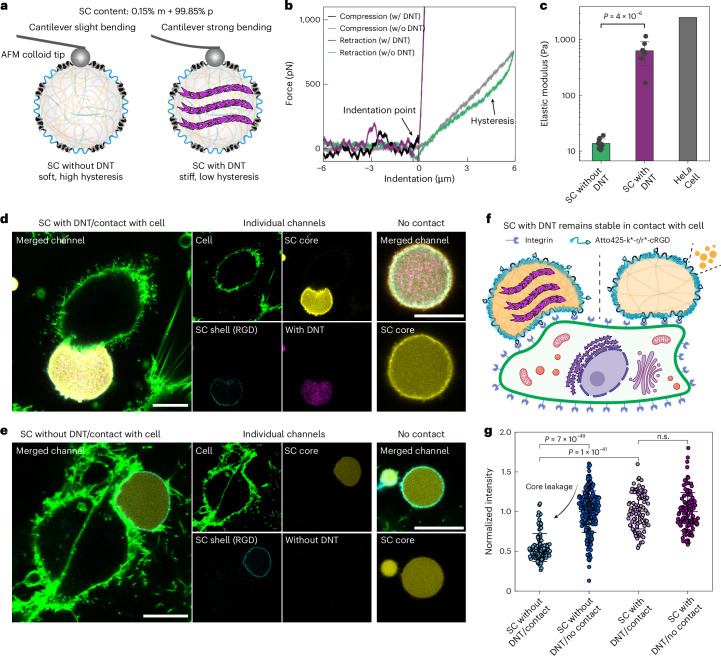
Artificial cytoskeleton enhances SC stiffness and stabilizes SC during SC–mammalian cell contact. **a**, A schematic of the SC stiffness probed by colloidal probe AFM. **b**, Representative force–distance curves in a full compression-retraction cycle for SCs with and without the DNT cytoskeletons. SCs with DNTs show elastic behavior without hysteresis, whereas SCs without DNTs show more viscous behavior with marked hysteresis in the compression/retraction cycle. **c**, The elastic moduli of SCs with and without DNT cytoskeletons (mean ± s.d., *n* = 8 SCs (with DNT) measured, *n* = 10 SCs (without DNT) measured), compared with HeLa cells reported in literature^63^. **d**, Representative CLSM images of SCs with DNTs in contact with HeLa cells and no-contact controls (cell membrane stained with CellMask). **e**, Representative CLSM images of SCs without DNTs in contact with HeLa cells and no-contact controls. High cell viability confirmed in Supplementary Fig. 18. **f**, A schematic of SC stability upon cell contact: SCs without DNTs destabilize and leak, whereas SCs with DNTs remain intact. **g**, A quantification of SC leakage with and without DNTs in contact with cell, measured by normalized SC core intensity (mean ± s.d., *n* > 90 SCs measured). CLSM channels: cell membrane (green), SC shell (cyan), SC core (yellow) and DNT (magenta). The DNTs were first grown in SCs at 20 °C. The SC-cell coincubation was performed at 25 °C with 5% CO_2_. Scale bars, 10 μm for **d** and **e**. n.s., not significant; w/, with; w/o, without. Source data

After establishing various control mechanisms for growing artificial cytoskeletons inside SCs, we further asked to what extent the DNT cytoskeletons indeed endow SCs with functional benefits regarding mechanical properties and stability. Studying the functional properties is an important aspect for transitioning the field of SCs from control over structure to functional molecular systems and, ultimately, applications. Similar to how cytoskeletons endow cells with mechanical strength^10^, the artificial cytoskeletons formed in our SCs also significantly enhance their elastic modulus by about two orders of magnitude compared with pristine SCs, as probed by atomic force microscopy (AFM) with a colloid probe^62^ (Fig. 6a–c). Interestingly, the compression-retraction force cycle for the pristine SCs without DNT cytoskeletons displays a hysteresis that signifies viscous energy dissipation and liquid-like properties. By contrast, SCs with DNT cytoskeletons exhibit largely elastic behavior without hysteresis.

The enhanced elastic modulus reaches the level of real cells reported in literature^63^, which opens possibilities for further exploring interactions between our SCs with real mammalian cells by building an interface to the mechanosensory system of cells. To this end, we synthesized an ssDNA-cRGD conjugate (r*-cRGD) based on our previously reported method and immobilized it to the SC shell via the k barcode using Atto425-k*-r/r*-cRGD binding^64^. cRGD is a peptide motif that can be recognized and bound by integrin of cells^65^. When incubating SCs with HeLa cells (green CellMask stain visualizes the plasma membrane), a marked deformation of SCs in contact with the cells can be observed, confirming a tight bonding as expected. This deformation is similar for both types of SC regardless of whether they contain a cytoskeleton or not (Fig. 6d,e). A slight tendency can be observed toward a bit more corrugated interface in absence of the cytoskeleton. However, while the cytoskeleton-containing SCs retain their content (p(A_20_-p)_*n*_), SCs without a cytoskeleton leak some of their interior. The leakage is significant and can be traced by a reduction of the yellow fluorescence in the interior that arises from the p(A_20_-p)_*n*_, labeled by Atto565-p*, that constitutes the majority of the SC core (Fig. 6d–g). This beneficial mechanical durability is because all artificial cytoskeletons grown within the SC are strongly bound to the core matrix, so that the mechanical property of these cytoskeletons can be tethered and coupled to the SC core. This represents a distinct functional benefit for building communicative SC–cell mechano-interfaces in the future.

## Discussion

We have introduced a versatile SC system that allows to study the assembly of subcellular-SC structures in viscoelastic environment, as exemplified for the formation of artificial cytoskeletons. We introduce co-phase separation as a molecular engineering approach to embed multiple orthogonal ssDNA barcodes inside the SCs to permit selective enrichment of self-assembling species. In contrast to classical crowding conditions, such as in dextran solutions^39^, the DNT building blocks and the arising DNTs remain tethered and hence coupled to the viscoelastic interior of the SCs. The growth of DNA tiles into extended fibers depends on the enriched tile concentration, and the process is much slower than in plain solution due to the coupling with the DNA polymer dynamics. On a mechanistic level, in contrast to classical crowding, bundling of the DNTs does not occur. In addition, the cytoskeleton growth rather proceeds by end-to-end joining of small fibrillar nuclei and not by tile addition commonly observed in solution^57–59^. We note that the current DNT assembly system used in our study only proceeds on a certain timescale of its own, and it can therefore also only couple to one particular timescale regime of the viscoelastic matrix (here, in our case, the terminal flow regime for uncrosslinked SCs). Nonetheless, by tuning the viscoelastic property of the SC matrix via crosslinking, temperature and concentration, we can modify the dynamics of the coupled SC matrix and influence the DNT assembly kinetics. In the future, it will be intriguing to explore how a more sophisticated assembly system, involving multiple kinetic processes across different timescales, would interact with various relaxation regimes of the viscoelastic environment and exhibit behaviors distinct from those observed in a simple solution.

With ternary barcode SC systems, different stimuli pathways and orthogonal tiles, we further realize metabolic growth of cytoskeleton inside SCs, as well as controlled formation and deconstruction of multiple artificial cytoskeletons inside the same SC. This is a step forward to mimic real cells that can organize multiple cytoskeletons in response to different stimuli or even in a metabolic fashion^35^. Moreover, we also show at a system level how SCs can selectively enrich desired monomers from a common pool in a diverse SC community to form distinct structures. Finally, we introduce a mechano-interface between SCs and living cells. Artificial cytoskeletons enhance the SC stiffness and lead to stability when in contact with real mammalian cells via a strong mechano-contact brought about by cRGD–integrin binding.

In a wider perspective, in contrast to classical SCs made by liposomes and polymersomes with plain aqueous compartments^25,39^ or coacervates with certain level of crowding^32,66,67^, our SC represents a step forward to mimic real cells featuring not only macromolecular crowding but also viscoelastic environment, programmable uptake and controlled local enrichment. These features are ubiquitous in biological and living systems but have been rare in SCs. Our SC can thus serve as a modular molecular systems engineering platform for studying self-assembly and structure formation under cell-like environments. In addition, our co-phase-separation approach endows SC systems with highly flexible programmable barcodes in their cytoplasm-mimetic matrix, which can be modularly utilized to design and functionalize SCs with higher degree of complexity and functionality. The selective enrichment behavior of our barcoded SCs in SC communities also opens simple pathways to establish communication, controlled targeting and programmable signaling. It can, for instance, be applied in DNA-based computing, circuits and diagnostics. Moreover, our SC–mammalian cell contact study also serves as a starting point for further exploration of SC interactions with biological systems. To summarize, we anticipate that this work provides a different perspective for understanding self-assembly processes in cell-mimicking environments, for the development of interactive SC protoecologies and for future investigation and design of SC–mammalian cell interactions.

## Methods

### Materials

ssDNAs were purchased from Biomers and Integrated DNA Technologies (IDT) (see Supplementary Data 2, oligonucleotide sequences, for a summary of all sequences used in this study. T4 DNA ligase (2 U μl^−1^), exonuclease I (40 U μl^−1^), exonuclease III (200 U μl^−1^) and Φ_29_ polymerase (10 U μl^−1^) were purchased from Lucigen. Thermal stable inorganic pyrophosphatase (2 U μl^−1^), AvaII restriction enzyme (10 U μl^−1^) and nuclease-free water were bought from New England BioLabs. Deoxynucleotide triphosphate (dATP, dTTP, dGTP and dCTP) (100 mM), aminoallyl-dUTP-ATTO-425 (1 mM), aminoallyl-dUTP-XX-ATTO-488 (1 mM), aminoallyl-dUTP-XX-ATTO-594 (1 mM) and aminoallyl-dUTP-ATTO-647N (1 mM) were purchased from Jena Bioscience. Hexadecane, sodium chloride, magnesium chloride, Tris(hydroxymethyl)-aminomethane hydrochloride (Tris–HCl, pH 8), Trizma base, acetic acid and ethylenediaminetetraacetic acid disodium salt dihydrate (EDTA) and fetal bovine serum (FBS) were purchased (as bioreagent grade if available) from Sigma-Aldrich. Agarose low electroendosmosis was purchased from AppliChem. RNase-free TE buffer (Invitrogen, 10 mM Tris and 1 mM EDTA, pH 8.0, 500 ml), RNase inhibitor (40 U μl^−1^), SYBR gold, GeneRuler 50 bp DNA Ladder, GeneRuler 1 kb DNA Ladder and Duke Standards dry borosilicate glass microspheres (18.2 ± 1 μm) were purchased from Thermo Fisher Scientific. The 96-well µ-plates were purchased from ibidi, and the 384-well high-content imaging glass bottom microplates were purchased from Corning. Tipless AFM probes cantilevers (HQ:NSC36/tipless/No Al) with three different AFM cantilevers with spring constants between 0.6 N m^−1^ and 2.0 N m^−1^ were purchased from MikroMasch. HeLa cell lines was obtained from Leibniz Institute DSMZ-German Collection of Microorganisms and Cell Cultures. Cyclo[Arg-Gly-Asp-d-Phe-Lys(PEG-PEG-azide)] (cRGDfK-N_3_; RGD-3759-PI) was purchased from Biosynth. Cell Eagle’s minimum essential medium (MEM), MEM-NEAA solution and phosphate-buffered saline were purchased from CARL ROTH. Penicillin–streptomycin and insulin-transferrin-selenium (ITS-G) were obtained from Gibco. CellMask green plasma membrane stain and Live/Dead viability/cytotoxicity assay kit (green/deep red) were purchased from Invitrogen.

### Instruments

All thermal annealing and heating ramps were performed on TPersonal Thermocycler (Analytik Jena) or Thermocycler Nexus GX2 (Eppendorf). Incubation was carried out on an Eppendorf ThermoMixer C. The DNA concentrations were determined by a ScanDrop (Analytik Jena) or a DS-11 Spectrophotometer (DeNovix). UV illumination was performed by BioLED Light Source Control Module (Mightex). CLSM was performed on a Leica Stellaris 5. SIM was performed on a ZEISS Elyra7 with Lattice SIM^2^. AFM colloid probes were fabricated by attaching glass beads to the tipless AFM cantilevers using a three-axis oil hydraulic micromanipulator (MMO-203 from Narishige) under a bright field optical microscope (Zeiss Axiotech). AFM measurements were performed using a JPK SPM NanoWizard III with a liquid sample cell on top of an inverted microscope stage (Zeiss Axiovert). The cells were cultured in MCO-170AIC CO_2_ Incubator (PHC Holdings Corporation). Cell viability experiments was performed on an EVOS M7000 (Invitrogen). Gel electrophoresis was performed in a gel chamber from Biostep.

### Preparation of DNA SCs via co-phase separation

The preparation of DNA SCs containing only p barcode is adapted from our previous reports with modifications for the formation of larger DNA SCs^19,54^ (Supplementary Fig. 1). p(A_20_-p)_*n*_ (0.5556 g l^−1^) and p(T_20_-k)_*n*_ (0.0694 g l^−1^) were mixed in TE buffer without any salt at a final volume of 9 μl. The solution mixture was then heated at 95 °C for 15 min (in addition to the first thermal cleavage during ssDNA synthesis mentioned above) for thermal cleavage to further reduce the chain length of the ssDNA polymers. Afterward, 1 μl of TE buffer containing 500 mM MgCl_2_ was introduced into the reaction mixture. The solution containing finally 0.5 g l^−1^ p(A_20_-p)_*n*_ and 0.0625 g l^−1^ p(T_20_-k)_*n*_ with 50 mM MgCl_2_ was heated to 95 °C for 20 min (to induce p(A_20_-p)_*n*_ phase separation) and subsequently cooled down to room temperature, during which the p(T_20_-k)_*n*_ localized as shell on the periphery of the p(A_20_-m)_*n*_ core by A_20_/T_20_ hybridization. The heating and cooling rates were both 3 °C s^−1^. Finally, the 10 μl solution containing the core–shell DNA SCs was diluted five times by adding 40 μl TE buffer containing various amounts of MgCl_2_ to reach desired salinity, for example, 15 or 50 mM of MgCl_2_. The obtained 50 μl DNA SCs solution (as 5× diluted) has 0.1 g l^−1^ p(A_20_-p)_*n*_ and 0.0125 g l^−1^ p(T_20_-k)_*n*_, corresponding to ~8 μM p barcode and ~1 μM k barcode, respectively, in total solution. The solution was then stored in a fridge at 4 °C for 1 week for equilibration. Before usage, the DNA SCs solution was always slightly vortexed to redisperse the DNA SCs homogenously in the solution.

To make SCs containing other barcodes (m, s, p and c) at different concentrations (from 400 to 40,000 nM), certain fraction of the p(A_20_-p)_*n*_ was replaced correspondingly by p(A_20_-m)_*n*_, p(A_20_-s)_*n*_ and p(A_20_-c)_*n*_, while maintaining the total concentration of p(A_20_-*x*)_*n*_ ssDNA (where *x* is m, s, p or c) constant at 0.5 g l^−1^. The successful integration of the desired barcodes in the SCs was then confirmed by CLSM (Figs. 2b and 3d).

The DNA polymers (cleaved for 30 min at 95 °C) used to prepare the DNA SCs were quantified by gel electrophoresis performed by 2 wt% agarose gel under 7 V cm^−1^ for 150 min in TAE buffer containing 40 mM Tris–HCl, 20 mM acetic acid and 1 mM EDTA (Supplementary Fig. 1). Post staining by SYBR gold was used to stain the DNA polymers. A quantification of chain length distribution and average chain length of DNA polymers was derived from the gel electrophoresis result based on previously reported method^68,69^.

### Preparation of DNA tiles and DNTs in solution

The DNA tiles design was adapted from previous reports with modifications^42^. The DNA tiles were prepared by heating the solution containing 5 μM of each of the needed strands (Supplementary Data 2) with 1× TAE buffer (40 mM Tris, 20 mM acetic acid and 1 mM EDTA) and 12.5 mM MgCl_2_ to 90 °C for 5 min and then cooling down to 20 °C (0.2 °C min^−1^) over a total time of ~6 h. The formed DNA tile solutions were stored at −20 °C for further experiments. To assemble DNTs of tile A and tile B in solution, 250 nM tile A and 250 nM tile B were mixed in 20 μl solution containing 1× TAE buffer and 12.5 mM MgCl_2_. During assembly at different time, 1 μl reaction mixture was taken and diluted into 10 μl solution containing 1× TAE buffer and 12.5 mM MgCl_2_ for CLSM imaging (Supplementary Fig. 3). To assemble DNAzyme-catalyzed DNTs of tile D in solution, 500 nM tile D, 5 μM DNAzyme and 8 U μl^−1^ RNase inhibitor were mixed in 20 μl solution containing 1× TAE buffer and 12.5 mM MgCl_2_. During assembly at different time, 2 μl reaction mixture was taken and diluted into 20 μl solution containing 1× TAE buffer and 12.5 mM MgCl_2_ for CLSM imaging.

### Formation of artificial cytoskeletons in SCs

For DNT formation by tile A and tile B in SCs with different m barcode concentrations (Fig. 2), 1 μl of 5× diluted SCs containing specific m barcode concentrations (at 15 mM MgCl_2_), 2 μl of 100 nM tile A and 2 μl of 100 nM tile B were added to 15 μl TE buffer containing 15 mM MgCl_2_ and gently mixed in a 200 μl transparent PCR tube. The solution mixture was then transferred to a 384-well plate and covered with 20 μl of hexadecane to prevent evaporation. The sample was incubated at different temperatures (20–35 °C) and imaged by CLSM over time.

For light-activated artificial cytoskeleton formation in SCs (Fig. 3a,b), 1 μl of the 5× diluted SCs (at 15 mM MgCl_2_) containing 400 nM m barcode and 2 μl of 100 nM tile C with a photo-cleavable hairpin were added to 17 μl TE buffer containing 15 mM MgCl_2_ and gently mixed in a 200 μl transparent PCR tube. The sample in the PCR tube was incubated at room temperature for roughly 30 min for tile enrichment and then illuminated with a 365 nm light-emitting diode (LED) light for 5 min with an intensity of 3.5 mW, before transferring to a well of a 384-well plate and subsequently covered with 20 μl of hexadecane to prevent evaporation. The sample was incubated at 20 °C and imaged by CLSM over time to track the temporal evolution of the formed artificial cytoskeletons inside SCs.

For DNAzyme-catalyzed artificial cytoskeleton formation in SCs (Fig. 3e,f), 1 μl of the 5× diluted SCs ([m] = 400 nM, [s] = 40,000 nM, at 15 mM MgCl_2_), 2 μl of 100 nM tile D, 2 μl of 40 U μl^−1^ RNase inhibitor, 0.9 μl of 800 nM DNAzyme were added to 14.1 μl TE buffer containing 15 mM MgCl_2_ and gently mixed in a 200 μl transparent PCR tube. The solution mixture was then transferred to a well of a 384-well plate and covered with 20 μl of hexadecane to prevent evaporation. The sample was incubated at 20 °C and imaged by CLSM over time.

For the formation of orthogonal artificial cytoskeletons inside the SCs (Fig. 4), 1 μl of the 5× diluted SCs (at 15 mM MgCl_2_) containing 400 nM m barcode and 400 nM s barcode, 2 μl of 100 nM tile C and 2 μl of 100 nM tile E were added to 15 μl TE buffer containing 15 mM MgCl_2_ and gently mixed in a 200 μl transparent PCR tube. For the light trigger, we applied 365 nm LED light illumination for 5 min with intensity of 3.5 mW. For the DSD trigger, we used 50 nM activator (2.5× over-stoichiometric to the inhibitor) to trigger the assembly of the tiles. The sample was incubated at 20 °C and imaged by CLSM over time to track the temporal evolution of the formed artificial cytoskeletons inside SCs. After 7 days of assembly, we added 100 nM deactivator (2× over-stoichiometric to the activator) to deconstruct the formed artificial cytoskeletons by tile E.

For selective formation of distinct artificial cytoskeletons inside individual SCs having different barcodes within an SC community (Fig. 5), 1 μl of each 5× diluted SCs containing different barcodes (blue-shell, magenta-shell, green-shell and yellow-shell SCs, at 15 mM MgCl_2_), 2 μl of 100 nM tile C and 2 μl of 100 nM tile E were added to 12 μl TE buffer containing 15 mM MgCl_2_ and gently mixed in a 200 μl transparent PCR tube. For the light trigger, we applied 365 nm LED light illumination for 5 min with intensity of 3.5 mW. For the DSD trigger, we used 50 nM activator (2.5× over-stoichiometric to the inhibitor) to trigger the assembly of the tiles. The sample was incubated at 20 °C and imaged by CLSM over time to track the temporal evolution of the formed artificial cytoskeletons inside different SCs.

### FRAP experiments

We performed FRAP experiment with a Leica Stellaris 5 on light-activated artificial cytoskeletons formed by tile A and tile B (0.1%, 1% and 10%) inside the SCs after 20 days of growth to probe dynamic properties of the artificial cytoskeletons (Supplementary Fig. 6). An SC containing artificial cytoskeletons labeled by Atto488 was first imaged with low laser intensity five times (2 s per frame) as prebleaching images, before ten times bleaching by 100% laser intensity in a rectangular region of interest (ROI) covering half of the SC to completely bleach the artificial cytoskeletons inside. Then, postbleaching images were manually recorded at different times over 48 h.

We also performed FRAP on various SCs (crosslinked, uncrosslinked, cleaved and so on) to probe the internal dynamics of the SCs (Supplementary Figs. 10, 12 and 13). SCs labeled by Atto488-m* attached to the m barcode were first imaged with low laser intensity five times (1 s per frame) as prebleaching images, before five times bleaching by 100% laser intensity in a circular ROI with a diameter of 2 μm inside the SC. Then, 120 frames of postbleaching images were recorded at a rate of 1 s per frame over 120 s. For quantification, we measured intensities within the circular ROI (*I*_ROI_) and intensities in a circular ROI away from bleached region within the SCs (*I*_ref_) in pre- and postbleaching images. We applied double normalization by ${I}_{{\rm{Norm}}}(t)=\frac{{I}_{{\rm{ROI}}}(t)}{{I}_{{\rm{ROI}}}({t}_{0})}\times \frac{{I}_{{\rm{ref}}}({t}_{0})}{{I}_{{\rm{ref}}}(t)}$, where *I*(*t*_0_) represents the intensity measured in the first image before bleaching and *I*(*t*) is the intensity measured over time.

### Reduction of DNA polymer concentration of SC matrix by enzymatic degradation

SCs containing 59.9% p barcode, 40% c barcode and 0.1% m barcode were prepared with the methods described above. To perform enzymatic degradation, 2 μl of such SCs (5× diluted, in TE buffer at 15 mM MgCl_2_) was mixed with 0.4 μl of 50 μM c* and 12.6 μl of TE buffer containing 15 mM MgCl_2_. After 1 h incubation for c/c* hybridization, 1 μl of AvaII enzyme (10 U μl^−1^) was added to enable sequence specific restriction at c/c* for another 1–2 h. The sample was then used to mix with 2 μl 100 nM tile A and 2 μl 100 nM tile B for growing artificial cytoskeletons inside the SCs.

### SIM measurements

SIM was conducted at an Elyra7 (ZEISS) using the proprietary ZEN black software for acquisition and data processing (Fig. 2f). SCs containing a 400 nM m barcode with formed DNTs inside were imaged in lattice SIM mode using the following parameters: objective: Plan-Apochromat 63×/1.4 Oil DIC M27. Beam splitters (primary, secondary): LBF 405/488/561/642, SBS BP 490–560 + LP 640. Magnifying lens: 1.6×. Laser: 642 nm diode, output: 0.5% (~400 µW). Exposure time: 100 ms per phase, 13 phases. SIM grating period: 32 µm. *Z*-stack: leap mode, *Z*-step 328.7 nm. Pixel size (*xy*): 62.6 nm. The raw data were processed using the ‘SIM’ processing function with the following parameters: Algorithm: SIM^2^ 3D Leap. Iterations: 20. Regularization weight: 0.08. Sampling rates (input and output): 2×. Sectioning: 100. Intensity: scale to raw.

### AFM measurements

Colloid AFM probes were fabricated by attaching borosilicate glass beads (18.2 ± 1 μm) to the tipless AFM cantilever end (spring constant 0.6 N m^−1^) with the help of micromanipulator under a bright field microscope. To perform AFM measurement on SC, we added SCs (99.85% p barcode, 0.15% m barcode) with and without DNT (formed via tile A and tile B) into an AFM liquid cell on a glass slide on top of the stage of the inverted microscope. The AFM colloid probe was then approached to the top of an SC under microscope. The force–distance measurement was then conducted in contact mode. The force spectroscopy data were fitted with the following equation, where *F* is the force, *E* is modulus, *ν* is Poisson’s ratio (we used 0.5 here), *R* is the effective radius calculated by harmonic mean of the radius of the SC (*R*_SC_) and the radius of the colloidal probe (*R*_probe_), that is, *R* *=* *R*_SC_ × *R*_probe_/(*R*_SC_ + *R*_probe_) and *d* is indentation distance

1 $$F=\frac{4}{3}\times \frac{E}{1-{\nu }^{2}}\times \sqrt{R}\times {d}^{1.5},$$

according to the Hertzian model (sphere–sphere geometry) to yield the modulus (Fig. 6a–c).

### SC–mammalian cell contact and cell viability assay

The HeLa cells were routinely cultured with complete MEM supplemented with 10% FBS (v/v), 1% MEM-NEAA and penicillin–streptomycin (v/v) in an incubator with 5% CO_2_ at 37 °C. We used click chemistry to conjugate cRGDfK-N_3_ to DBCO-r* for obtaining cRGD-r* based on our reported method^64^. cRGD-r* was further annealed with Atto425-k*-r to form Atto425-k*-r/r*-cRGD complex by heating up to 90 °C for 3 min (3 °C s^−1^), which was then slowly cooled down to 20 °C (0.02 °C s^−1^). SCs with DNT inside were labeled on their shell by mixing SCs with Atto425-k*-r/r*-cRGD complex. The ratio between Atto425-k*-r/r*-cRGD and the k barcode in the SC shells was always kept at 80% to avoid free cRGD in solution.

For the SC–mammalian cell contact experiment, the HeLa cells were first incubated in a 96-well plate with complete MEM culture medium (10% FBS) for 15 h for adhering to the well surface, then the complete medium was replaced with adapted MEM medium which contains 5% FBS, 1× ITS-G, 100 μg ml^−1^ actin and 12.5 mM MgCl_2_. Then, we added SCs with cRGD on the shell to the prepared HeLa cells in the well for coincubation at 25 °C with 5 % CO_2_ for 3 h. Before CLSM study, the cells were stained by CellMask green plasma membrane dye (500× diluted) for 10 min.

To verify cell viability during coincubation with SCs, we prepared independent HeLa cells with the same method described above. We then performed Live/Dead Viability/Cytotoxicity Assay with 2,000× dilution factor for the Calcein, AM and SYTOX Deep Red Nucleic Acid Stain, according to the manufacturer’s instructions, and imaged the cells under CLSM and EVOS microscope (Supplementary Fig. 18).

### Statistical analysis

All data points featuring statistical analysis are presented as mean ± standard deviation (s.d.). The sample size (*n*) for each statistical analysis is reported in the relevant figure legend. The *P* values are calculated by an analysis of variance test (single factor). Statistical analysis was carried out using Origin 2023 and Microsoft Excel.

### Reporting summary

Further information on research design is available in the Nature Portfolio Reporting Summary linked to this article.

## Supplementary information

Supplementary Information Supplementary Figs. 1–19, Notes 1 and 2, Method 1, Table 1 and references.

Reporting Summary

Supplementary Data 1 Source data for Supplementary Information.

Supplementary Data 2 Oligonucleotide sequences used in this study.

Supplementary Video 1 Artificial cytoskeletons have different dynamic properties inside SCs at different temperatures.

## Source data

Source Data Fig. 2 Statistical source data.

Source Data Fig. 6 Statistical source data.

## Data Availability

All data are available in the article and its Supplementary Information.
