# Supplementary material for: Growing functional artificial cytoskeletons in the viscoelastic confinement of DNA synthetic cells
Source: Nat Chem Eng. 2025 Oct 7;2(10):627–39. doi: 10.1038/s44286-025-00289-5 (PMC12545198; doi:10.1038/s44286-025-00289-5)
Supplement: Supplementary file 1 — Supplementary Figs. 1–19, Notes 1 and 2, Method 1, Table 1 and references. [file 44286_2025_289_MOESM1_ESM.pdf]

nature chemical engineering

Supplementary information https://doi.org/10.1038/s44286-025-00289-5

Growing functional artificial cytoskeletons in the viscoelastic confinement of DNA synthetic cells

In the format provided by the authors and unedited

Table of Contents

Supplementary Note 1. Estimation of total barcode concentration in SCs. 3

Supplementary Note 2. Calculation of overlap concentration (c*) and entanglement concentration (c_e). 4

Supplementary Method 1. Synthesis of circular ssDNA templates and long ssDNA polymers 6

Supplementary Fig. 1: Synthesis of ssDNA multiblock copolymers by rolling circle amplification (RCA) and formation of synthetic cells by liquid-liquid phase separation. 7

Supplementary Fig. 2: Determination of total barcode concentration in DNA SC. 8

Supplementary Fig. 3: Complementary tile A and tile B form DNA nanotubes (DNTs) with low degree of branching in plain solution.. 9

Supplementary Fig. 4: Excess of DNA tiles allows enrichment of tiles inside the SCs through hybridization at the barcodes. 10

Supplementary Fig. 5: No significant partitioning of tiles without barcodes into SCs. 11

Supplementary Fig. 6: Dynamics of DNTs inside SCs by fluorescence recovery after photobleaching (FRAP) measurements. 12

Supplementary Fig. 7: Increase of temperature enables the formation of DNTs in SCs with high [m]... 13

Supplementary Fig. 8: Additional CLSM images showing the consistency in the formed structures of artificial cytoskeletons across individual SCs... 14

Supplementary Fig. 9: Unbound DNA tiles in solution contribute only marginally to the growth of DNTs inside SCs. 15

Supplementary Fig. 10: Crosslinking prevents cytoskeleton assembly....................................... 16

Supplementary Fig. 11: Representative CLSM image showing bundling of DNTs in a concentrated $p(A_{20}\text{-}p)_n$ solution. 17

Supplementary Fig. 12: Reduction of DNA polymer concentration of SC matrix accelerates the DNT assembly inside SCs. 18

Supplementary Fig. 13: Formation of artificial cytoskeletons insides SCs at different temperatures.. 20

Supplementary Fig. 14: Light-activated DNT assembly using photocaged DNA tile C............. 21

Supplementary Fig. 15: Control for light-activated artificial cytoskeleton inside SC shows absence of assembly.. 22

Supplementary Fig. 16: DNAzyme-catalyzed DNT assembly using DNA tile D in plain solution. 23

Supplementary Fig. 17: DSD-activated DNT assembly using DNA tile E. 24

Supplementary Fig. 18: Cell viability after co-incubation with SCs. 25

Supplementary Fig. 19. Uncropped gel scans.. 26

Supplementary Table 1. Details about composition of SCs with varied internal barcode concentrations, barcode concentration in solution, DNA tile concentration in solution, and stoichiometry between DNA tile and barcode in individual experiments. 27

References.. 28

Supplementary Note 1. Estimation of total barcode concentration in SCs.

In Supplementary Fig. 2, we determined the local concentration of total barcode inside SCs to be 850 μM in TE buffer containing 50 mM Mg^{2+}. Given a roughly 2.2 times volumetric swelling after lowering the salinity from 50 mM Mg^{2+} to 15 mM Mg^{2+} (Supplementary Fig. 2e), the total barcode concentration inside the SCs is about 400 μM, which is relevant to the salinity used throughout this study and corresponds to 100% of barcode inside SCs.

The molecular weight of the repeating unit of $p(A_{20}\text{-}x)_n$ (x = p, m, or s) is in the range of 12600 – 13000 g/mol depending on the barcode. Therefore, 400 μM barcode in the SC core corresponds to a local concentration of 5.04 – 5.20 g/L.

For SCs made by mixture of adenine-rich DNA polymers having different barcodes, the concentration of a specific barcode inside the SCs scales with the fraction of this particular barcode used in the mixture. For instance, 10% of m barcode corresponds to 40 μM, 1% of m barcode corresponds to 4 μM, and 0.1% of m barcode corresponds to 400 nM, etc.

Supplementary Note 2. Calculation of overlap concentration (c^*) and entanglement concentration (c_e).

In our recent publication, we have used MD simulations to obtain the end-to-end distance and radius of gyration of a DNA polymer with 1025 nt (end-to-end distance, R_e = 58.3 nm, radius of gyration R_g = 23.3 nm)[1].

Assuming a good solvent scenario, $R_e \sim R_g \sim N^{0.588}$ and taking 3 different DNA polymer lengths, which are characteristic of our DNA polymer size distribution (see Supplementary Fig. 1e, 3,000 nt as average molecular weight, 10,000 nt and 20,000 nt for long polymers present that have a strong influence on the matrix properties), we can first calculate the end-to-end distance (R_e) and radius of gyration (R_g) for three DNA polymer lengths:

R_e: 3,000 nt = $(3000/1025)^{0.588} \times 58.3$ = 109.63 nm.

10,000 nt = $(10000/1025)^{0.588} \times 58.3$ = 222.52 nm.

20,000 nt = $(20000/1025)^{0.588} \times 58.3$ = 334.49 nm.

R_g: 3,000 nt = $(3000/1025)^{0.588} \times 23.3$ = 43.81 nm.

10,000 nt = $(10000/1025)^{0.588} \times 23.3$ = 88.93 nm.

20,000 nt = $(20000/1025)^{0.588} \times 23.3$ = 133.68 nm.

The overlap concentration (c^*) can be estimated based on

$$c^* = \frac{3M}{N_A 4\pi R^3}$$

where M is the molecular weight and N_A is the Avogadro constant.

Each repeating unit has a molecular weight (on average) of 313 Da.

As it is debated whether to use R_e or R_g for calculating c^*, here we calculate both for obtaining the lower and upper limit of c*[2].

Applying R_e yields the **lower limit** of c^*:

c^*_{lower} (based on R_e): 3,000 nt = 0.28 g/L.

10,000 nt = 0.11 g/L.

20,000 nt = 0.066 g/L.

Applying R_g yields the **upper limit** of c^*:

c^*_{upper} (based on R_g): 3,000 nt = 4.43 g/L.

10,000 nt = 1.77 g/L.

20,000 nt = 1.04 g/L.

All calculations indicate the SC with 5 g/L to be above the overlap concentration, c*.

For completion, we also discuss the entanglement concentration. As the **entanglement concentration** (c_e) is typically 10 times the overlap concentration[2], the calculated c^* has to be multiplied by 10 to reach c_e, yielding:

$c_{e,\,lower}$: 3,000 nt ≈ 2.8 g/L.

10,000 nt ≈ 1.1 g/L.

20,000 nt ≈ 0.66 g/L.

$c_{e,\,upper}$: 3,000 nt ≈ 44.3 g/L.

10,000 nt ≈ 17.7 g/L.

20,000 nt ≈ 10.4 g/L.

This indicates the SC matrix to be with *conservative estimate* on the order of c_e.

Supplementary Method 1. Synthesis of circular ssDNA templates and long ssDNA polymers

Synthesis of circular DNA template and its corresponding ssDNA polymer is adapted from our previous reports (Supplementary Fig. 1)[3,4]. The linear ssDNA template and the corresponding ligation strand were firstly mixed at concentration of 1 μM in 100 μL TE buffer containing 100 mM NaCl. The solution was heated to 85 °C (3 °C/s) for 5 min before cooling to 25 °C (0.01 °C/s) for complete hybridization. Afterwards, 20 μL of 10× Ligase buffer (500 mM Tris-HCl, 100 mM $MgCl_2$, 50 mM dithiothreitol and 10 mM ATP (Lucigen)), 70 μL of nuclease-free water and 10 μL of T4 DNA Ligase (2 U/μL (Lucigen)) were introduced into the reaction mixture and gently mixed before leaving at room temperature for 3 h. The reaction mixture was then heated to 70 °C for 20 min to deactivate the enzyme. Then, 10 μL of Exonuclease I (40 U/μL (Lucigen)) and 10 μL of Exonuclease III (200 U/μL (Lucigen)) were added into the reaction mixture to react overnight at 37 °C for degradation of the ligation strands and any non-circularized templates in solution. Afterwards, the reaction mixture was heated to 80 °C for 40 min to deactivate the enzymes. To obtain the final circular ssDNA templates, the reaction mixture was washed by adding 400 μL TE buffer and filtrated using Amicon Ultra-centrifugal filters with a 10 kDa cut-off (Merck Millipore) for three times. The concentrations of the collected circular ssDNA templates were measured by the DS-11 Spectrophotometer (DeNovix), and the templates were stored in TE buffer at -20 °C.

For synthesis of long ssDNA polymers, we used rolling circle amplification (RCA). 5 μL of circular template (1 μM in TE buffer) and 1 μL of exonuclease resistant primer (10 μM in TE buffer) were mixed with 76 μL nuclease-free water, 10 μL of commercial 10× polymerase buffer (500 mM Tris-HCl, 100 mM $(NH_4)_2SO_4$, 40 mM dithiothreitol, 100 mM $MgCl_2$ (Lucigen)), 2 μL of Φ_{29} DNA polymerase (10 U/μL (Lucigen)), 1 μL of thermal stable inorganic pyrophosphatase (2 U/μL (NEB)) and 5 μL of adjusted deoxyribose nucleoside 5'-triphosphate mix (100 mM, the mix contains pure dATP, dTTP, dCTP, and dGTP solutions mixed in corresponding proportions of the exact composition of the desired ssDNA polymer repeating units (Jena Bioscience)). Note that for synthesis of ssDNA polymers with in-chain fluorophores, we further add 0.017 mM dUTP with desired fluorescent labels (aminoallyl-dUTP-ATTO-425, aminoallyl-dUTP-XX-ATTO-488, aminoallyl-dUTP-XX-ATTO-594, or aminoallyl-dUTP-ATTO-647N) for random insertion of the dye along the ssDNA chains during RCA. The reaction mixture was kept at 30 °C for 50 – 60 h before thermal cleavage at 95 °C for 15 min to shorten the ultrahigh molecular weight ssDNA. The final products were purified by rinsing with 400 μL TE buffer and filtration in Amicon Ultra-centrifugal filters with 30 kDa cut-off (Merck Millipore) for three times. The concentrations of the collected final ssDNA polymers were measured using the DS-11 Spectrophotometer (DeNovix), and the DNA polymers (cleaved at 95 °C for 15 min) were stored in TE buffer at -20 °C.

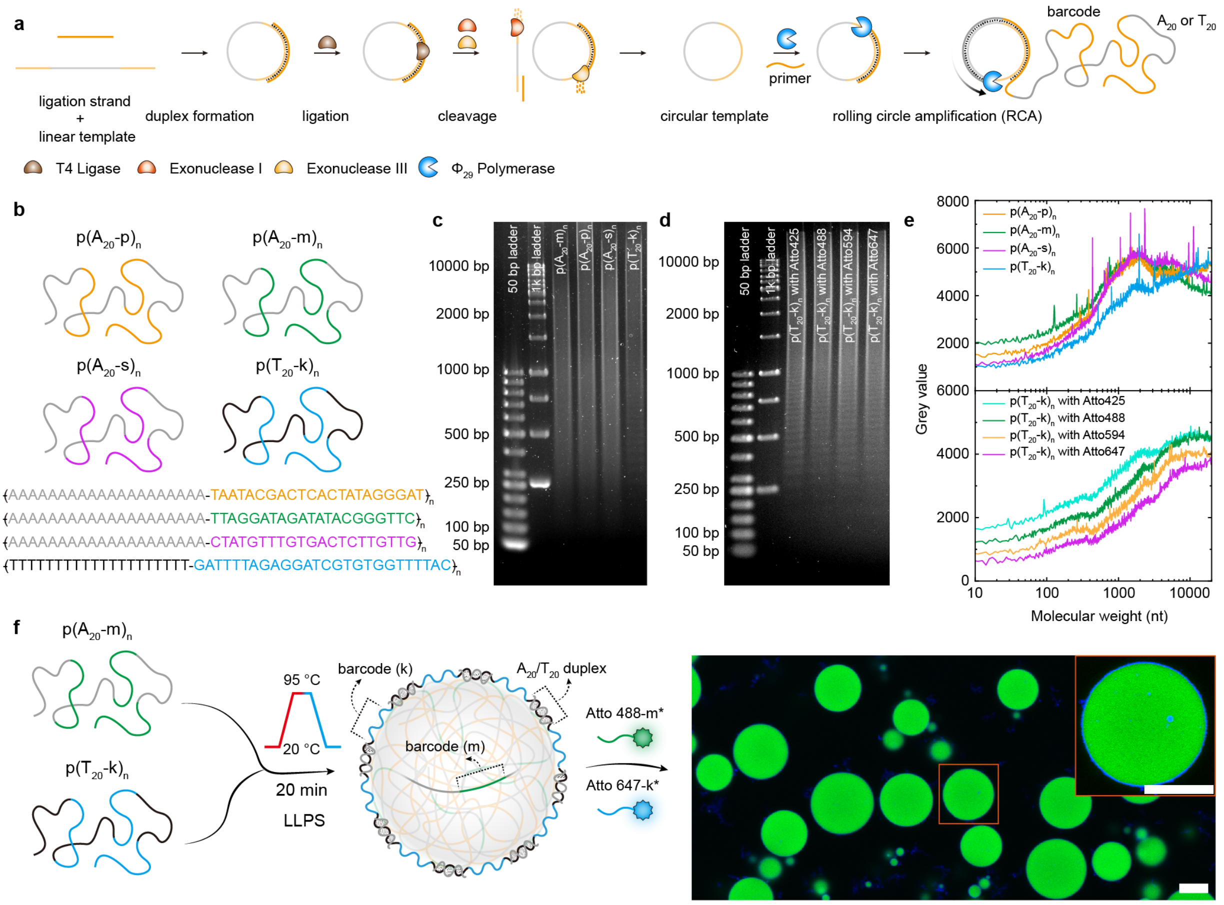

Supplementary Fig. 1: Synthesis of ssDNA multiblock copolymers by rolling circle amplification (RCA) and formation of synthetic cells by liquid-liquid phase separation.

a, Schematic representation for the synthesis of DNA polymers including preparation of circular template and RCA. **b**, DNA polymers synthesized and used in this work and their sequences. **c**, Representative gel electrophoresis of the generally used adenine-rich and thymine-rich DNA polymers, which are thermally heated at 95 °C for 30 min and used to make SCs. **d**, Gel electrophoresis of p(T_{20}-k)$_n$ containing different in-chain fluorophores, which are thermally heated at 95 °C for 30 min and used to make SCs. **e**, Quantification for the molecular weight distribution of DNA polymers shown in **c** and **d**. The molecular weight is reported as nucleotide number (nt), corresponding to double of the calibrated base pair number (bp). **f**, Scheme and representative CLSM image for the formation of SCs by LLPS of DNA polymers, resulting in core-shell SCs with addressable barcodes in the core and at the shell, which are labeled by Atto488-m* (green channel) and Atto647-k* (blue channel). The inset shows a zoomed-in SC corresponding to the orange box with visible shell. Uncropped gel scans for **c** and **d** can be found in Supplementary Fig, 19. Scale bars, 10 μm (**f**).

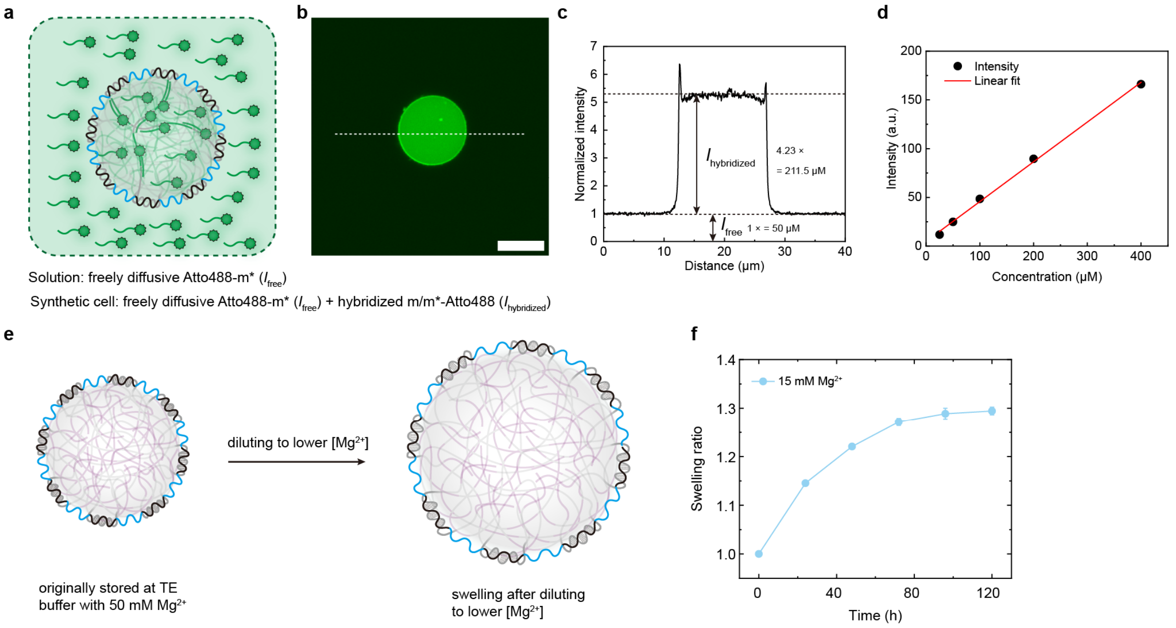

Supplementary Fig. 2: Determination of total barcode concentration in DNA SC.

a, Schematic representation of the titration experiment for determining the total barcode concentration in DNA SC. DNA SCs with 100% m barcode were mixed with high concentrations of Atto488-m* (50 μM) with a total concentration of m barcodes in the solution at 0.4 μM. Within the DNA SCs, all m barcodes are hybridized by Atto488-m*, and the SC is in a swollen state. The fluorescence intensity in the solution is only due to freely diffusing Atto488-m*, yielding I_{free}. In the SC, there are both hybridized Atto488-m* and freely diffusing Atto488-m*, giving $I_{hybridized} + I_{free}$. The intensity ratio ($I_R$) inside and outside the SC is then $(I_{hybridized} + I_{free})/I_{hybridized}$. **b**, Representative CLSM image of a SC in solution containing 50 μM Atto488-m*. **c**, Corresponding cross-sectional line profile along the dashed line in **b**. The intensity is normalized to the solution intensity, *i.e.*, divided by I_{free}. Thus, normalized intensity = 1 means the intensity contributed from freely diffusive Atto488-m*, while the additional contributions above 1 within a DNA SC stem from the hybridized Atto488-m*, $I_{hybridized}$. As the solution contains 50 μM Atto488-m*, given the intensity ratio in and outside SC, $(I_{hybridized} - I_{free})/I_{free}$ = 4.23, the SC therefore contains 50 μM × 4.23 = 211.5 μM (Note that a linear correlation between fluorescence intensity and fluorophore concentration is verified in **d**), reflecting the concentration of m barcode within the SCs after hybridization and swelling. Based on a roughly 4-fold swelling ratio in volume after m/m* hybridization, the barcode concentration can be calculated to be ca. 850 μM in the non-hybridized pristine SCs. **d**, Linear correlation between fluorescence intensity and Atto488-m* concentration. **e**, Schematic representation of the swelling of the SC induced by diluting the salinity (Mg^{2+}) in the solution. **f**, Swelling kinetics of the SCs after diluting to [Mg^{2+}] = 15 mM. The SCs are in equilibrium swollen state without further swelling after ca. 120 h (mean ± s.d., n = 5 SCs measured). Experiment temperature = 20 °C. Scale bar, 10 μm (**b**).

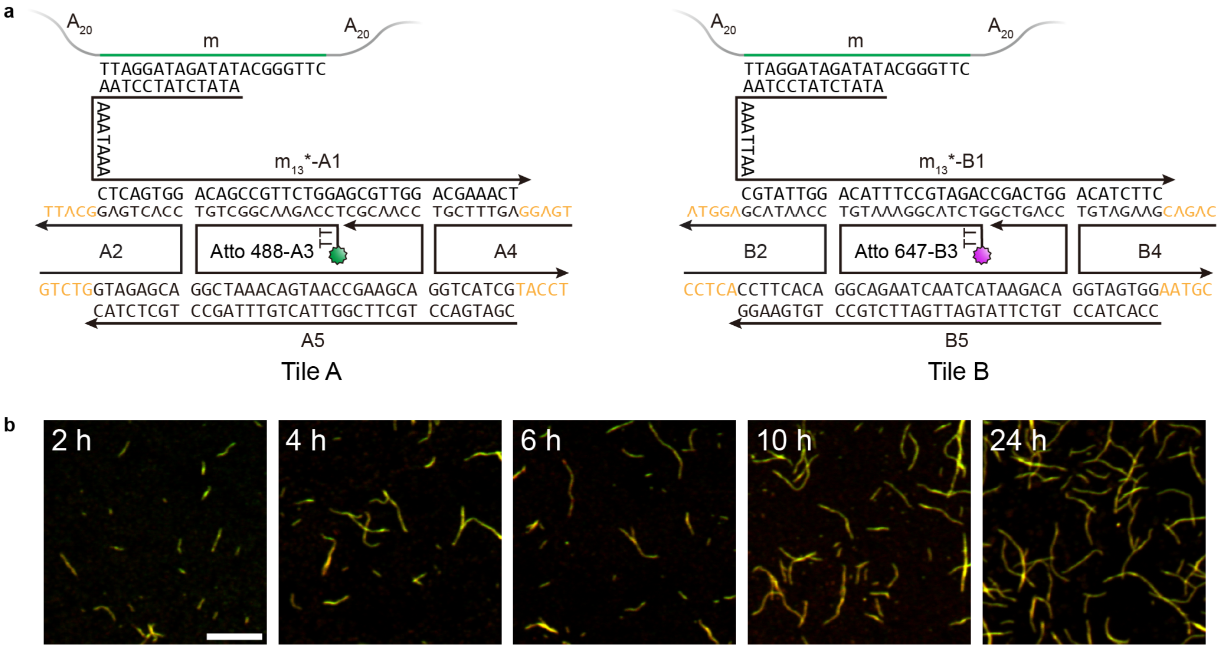

Supplementary Fig. 3: Complementary tile A and tile B form DNA nanotubes (DNTs) with low degree of branching in plain solution.

a, Design of the mutually complementary DNA tiles, tile A and tile B. Both contain a dangling strand partially complementary to a part of the m barcode of p(A_{20}-m)$_n$. Tile A contains Atto488 and tile B contains Atto647 as fluorescent labels. **b**, Time series CLSM images showing the formation of DNTs in solution over time. Experiment temperature = 20 °C. Scale bar, 5 µm (**b**).

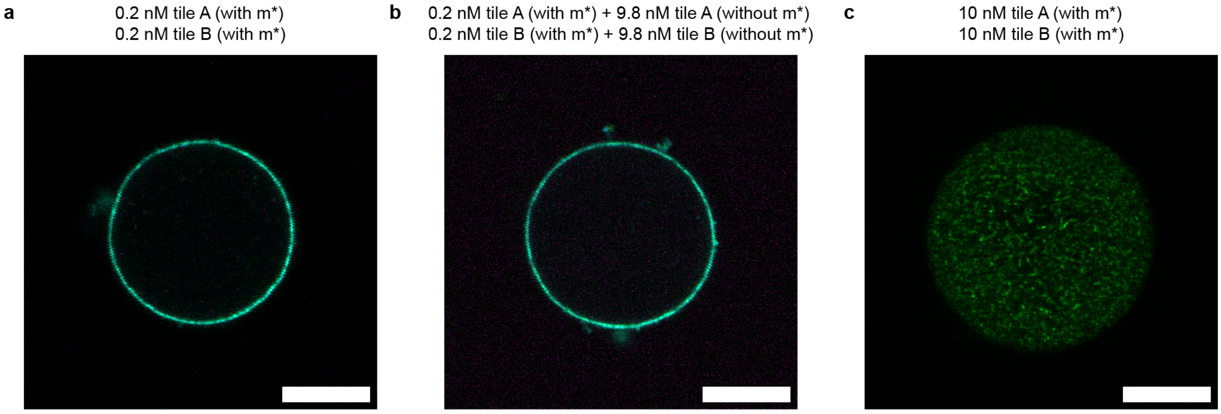

Supplementary Fig. 4: Excess of DNA tiles allows enrichment of tiles inside the SCs through hybridization at the barcodes.

a, Representative CLSM image of a SC incubated with 0.2 nM tile A (labeled by Atto488, green channel) and 0.2 nM tile B (labeled by Atto647, magenta channel), both containing a barcode m*, shows no enrichment of tiles inside the SCs (100 h incubation time, 20 °C, SC shell is labeled by Atto390-k* (cyan channel)). **b**, Representative CLSM image of a SC incubated with 0.2 nM tile A (labeled by Atto488, green channel) and 0.2 nM tile B (labeled by Atto647, magenta channel), both with barcode m*, as well as 9.8 nM tile A (labeled by Atto488, green channel) and 9.8 nM tile B (labeled by Atto647, magenta channel) without barcode m* shows no enrichment of tiles inside the SCs (100 h incubation time, 20 °C, SC shell is labeled by Atto390-k* (cyan channel)). **c**, Representative CLSM image of a SC incubated with 10 nM tile A (labeled by Atto488, green channel) and 10 nM tile B (labeled by Atto647, magenta channel), both with barcode m*, shows enrichment and initial assembly of DNA tiles inside the SC (100 h incubation time, 20 °C, SC shell is not labeled). Only green channel is shown in **c** for clarity. Scale bars, 10 µm (**a**, **b**, **c**).

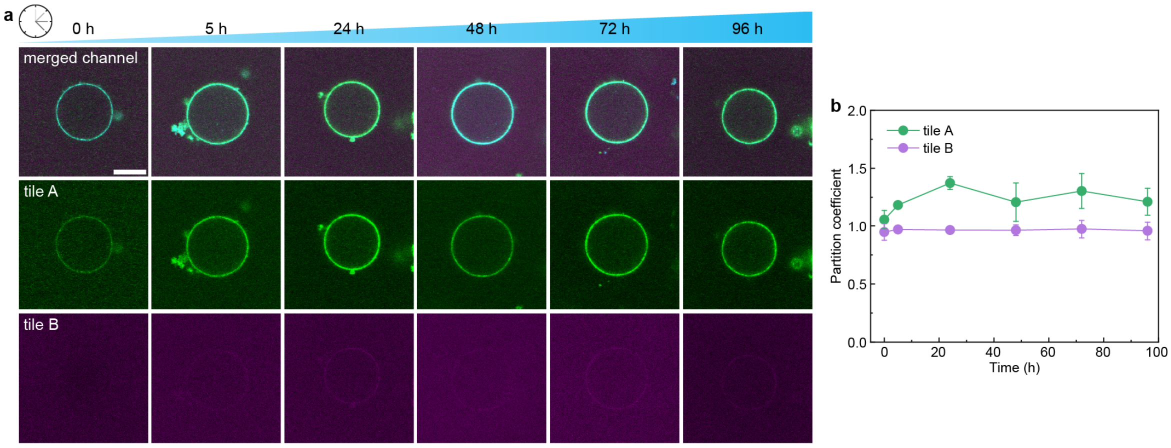

Supplementary Fig. 5: No significant partitioning of tiles without barcodes into SCs.

a, Representative CLSM images showing the distribution of tile A (Atto488, green channel) and tile B (Atto647N, magenta channel) across SCs (labeled at shell by Atto390-k*, cyan channel) and background solution over 96 h. Both tiles do not have a barcode interaction (m/m*) as otherwise used throughout this study. **b**, Partition coefficient of tile A and tile B as a function of time. The partition coefficient is quantified by the ratio between mean intensity inside the SCs and outside the SCs. The partition coefficients of both tiles are near 1, indicating a rather even distribution of tiles across SCs and solution (mean ± s.d., $n = 3 - 4$ SCs measured). Experimental temperature = 20 °C. Scale bar, 10 µm (**a**).

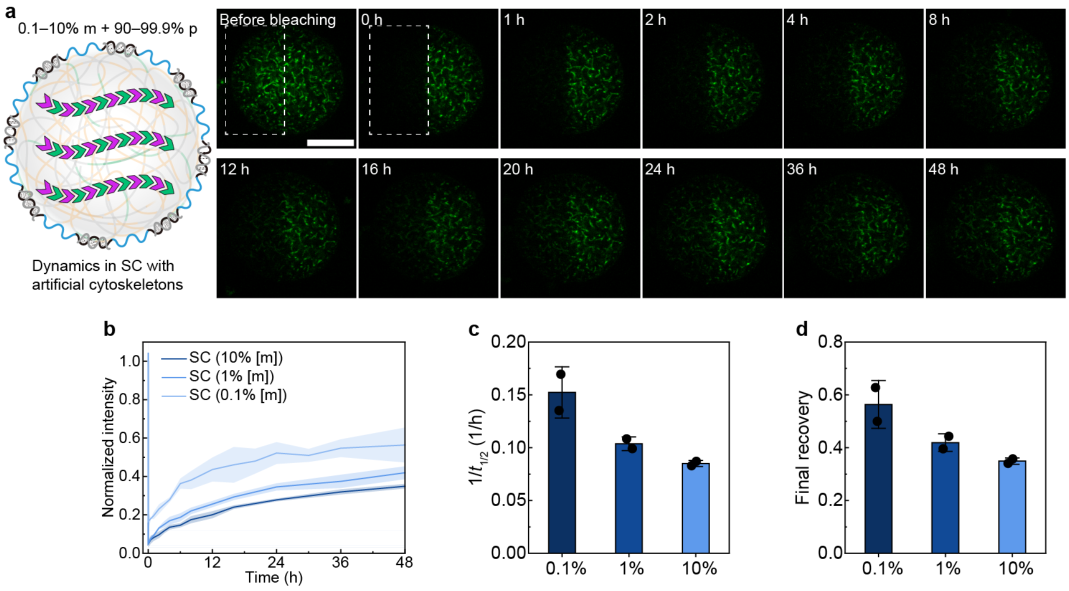

Supplementary Fig. 6: Dynamics of DNTs inside SCs by fluorescence recovery after photobleaching (FRAP) measurements.

a, Scheme for a SC with DNTs inside and representative time-dependent CLSM images of an SC with 0.1% DNTs inside, showing the FRAP (white dashed box) at different time points, revealing rather slow dynamics. **b**, Normalized intensity in the bleached area as a function of time for half-bleaching FRAP experiments performed on DNTs inside SCs after 14 days of assembly at different concentrations from 0.1% to 10% ([m] = 400 nM – 40000 nM inside SC, mean ± s.d., $n = 2$ FRAP experiments at each concentration). **c**, Half recovery time extracted from the FRAP data in **b** for DNTs inside SCs at different concentrations (mean ± s.d., $n = 2$ FRAP experiments at each concentration). **d**, Final recovery percentage extracted from the FRAP data in **b** for DNTs inside SCs at different concentrations (mean ± s.d., $n = 2$ FRAP experiments at each concentration). Recovery is faster and more complete for lower degree of loading of DNTs. Experiment temperature = 20 °C. Scale bar, 10 µm (**a**).

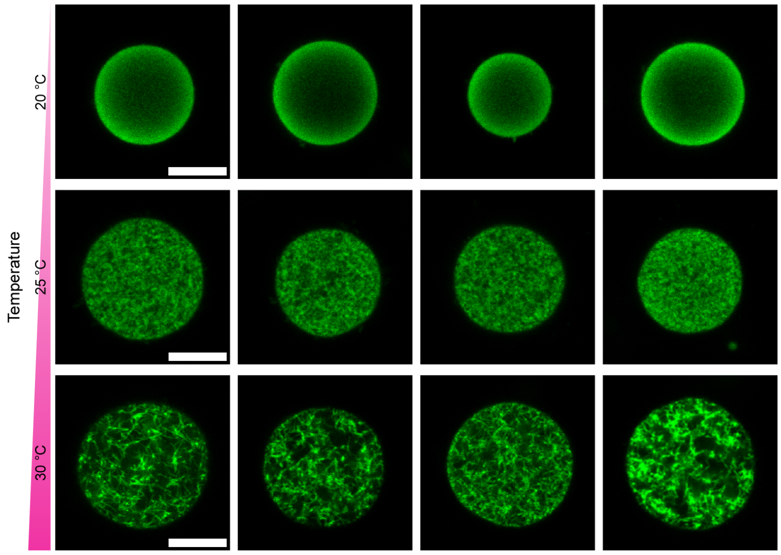

Supplementary Fig. 7: Increase of temperature enables the formation of DNTs in SCs with high [m].

Representative CLSM images showing DNA tile assembly inside SCs ([m] = 40,000 nM) after 2 days at different temperatures. At 20 °C, DNT formation is absent inside SCs. At 25 °C, there is a high density of DNA tiles inside, with a certain level of assembly into short DNTs. At 30 °C, the DNTs inside the SCs are discernible and yield a network structure. The assembly at higher temperature is possible due to enhanced dynamics of the SC matrix and weaker m/m* interactions. Scale bars, 10 µm.

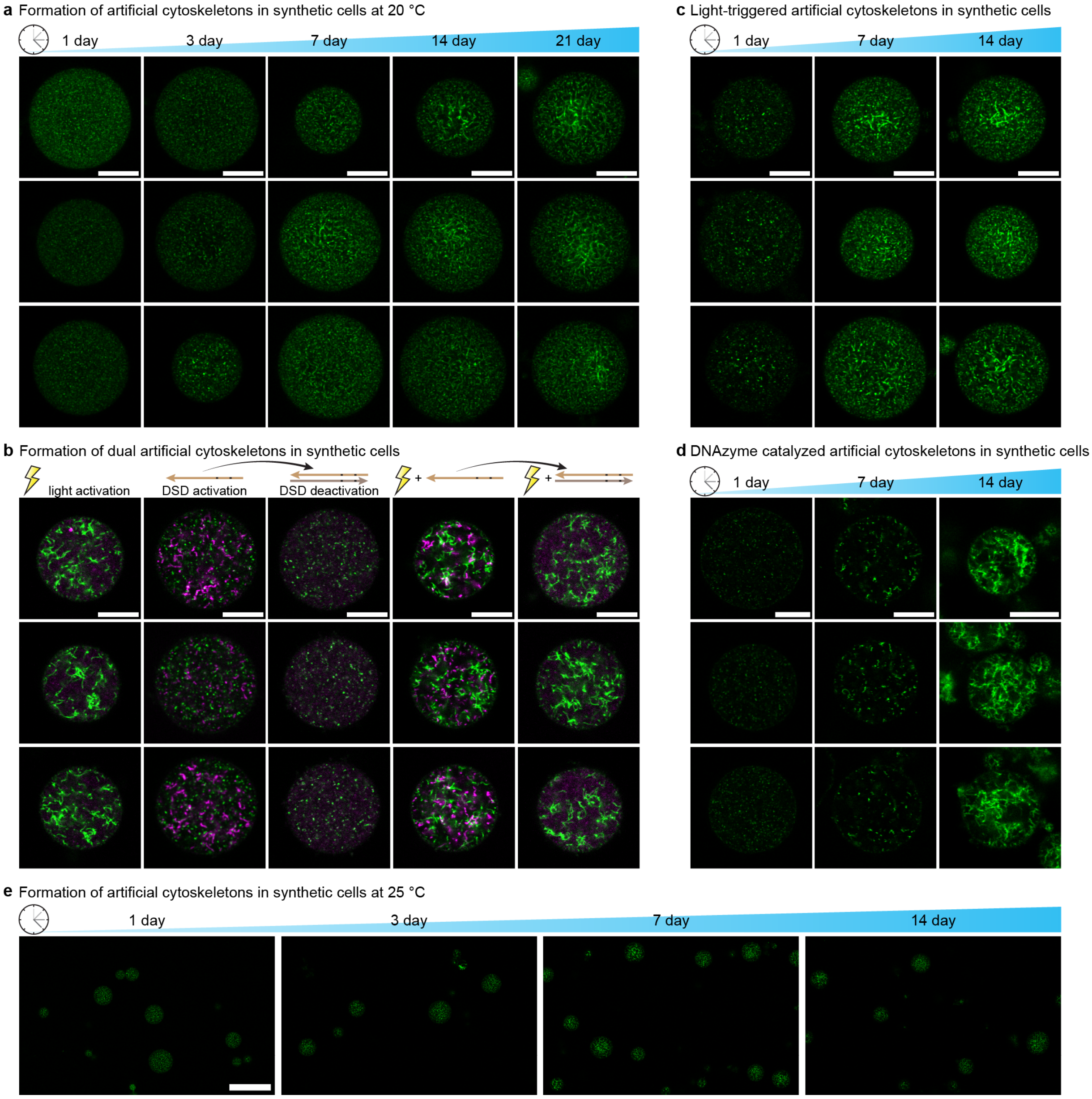

Supplementary Fig. 8: Additional CLSM images showing the consistency in the formed structures of artificial cytoskeletons across individual SCs.

a, CLSM images showing consistent structures for the formation of artificial cytoskeletons in SCs over time for Extended Data Fig. 2. **b**, CLSM images showing consistent structures for the formation of dual artificial cytoskeletons in SCs enabled by light and DSD activation for Fig. 4b. **c**, CLSM images showing consistent structures for the light-triggered artificial cytoskeletons in SCs for Fig. 3b. **d**, CLSM images showing consistent structures for the DNAzyme catalyzed artificial cytoskeletons in SCs for Fig. 3f. **e**, CLSM images showing several SCs with consistent structures of artificial cytoskeletons during their growth over time at 25 °C. Scale bars, 10 μm (**a**–**d**), 40 μm (**e**). We refer to the corresponding figures in the main text for details on the conditions.

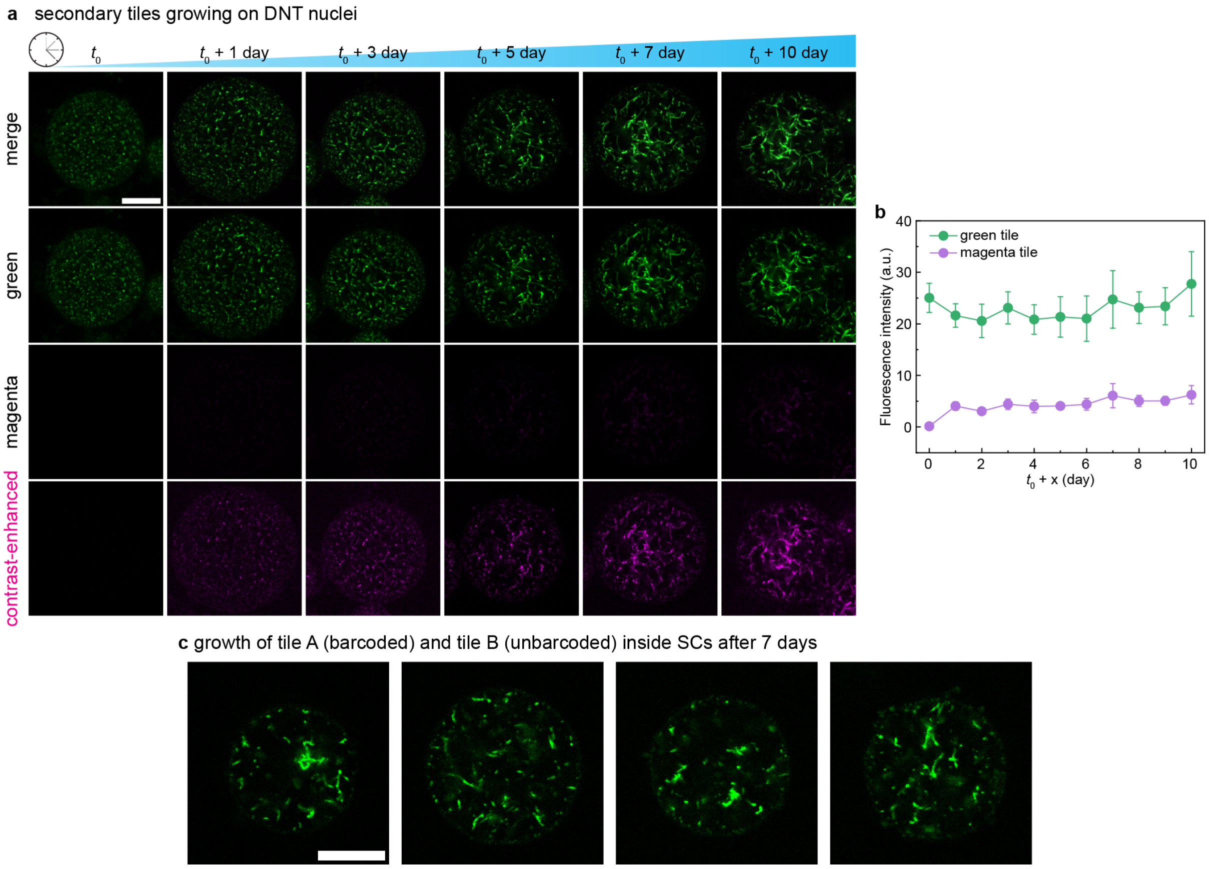

Supplementary Fig. 9: Unbound DNA tiles in solution contribute only marginally to the growth of DNTs inside SCs.

We firstly incubated SCs with green DNA tiles (10 nM tile A and 10 nM tile B) for 2 days, allowing them to grow into nuclei stage. After 2 days (t_0), we then added magenta tiles (10 nM tile A and 10 nM tile B) to see if they can co-assemble with the existing DNT nuclei.

a, Representative CLSM images and contrast-enhanced CLSM images in the magenta channel in the fourth row visualize a marginal integration of the magenta tiles. **b**, Fluorescence intensity of green tile and magenta tile measured inside the SCs at different times (mean ± s.d., n = 12 – 16 SCs measured at each day). **c**, Further control: Representative CLSM images showing that the growth of barcoded tile A and unbarcoded/not immobilized tile B after 7 days results in ill-defined, short DNT structures, and a lack of mature network structure inside the SCs. Experimental temperature = 20 °C. Scale bars, 10 µm (**a**, **c**).

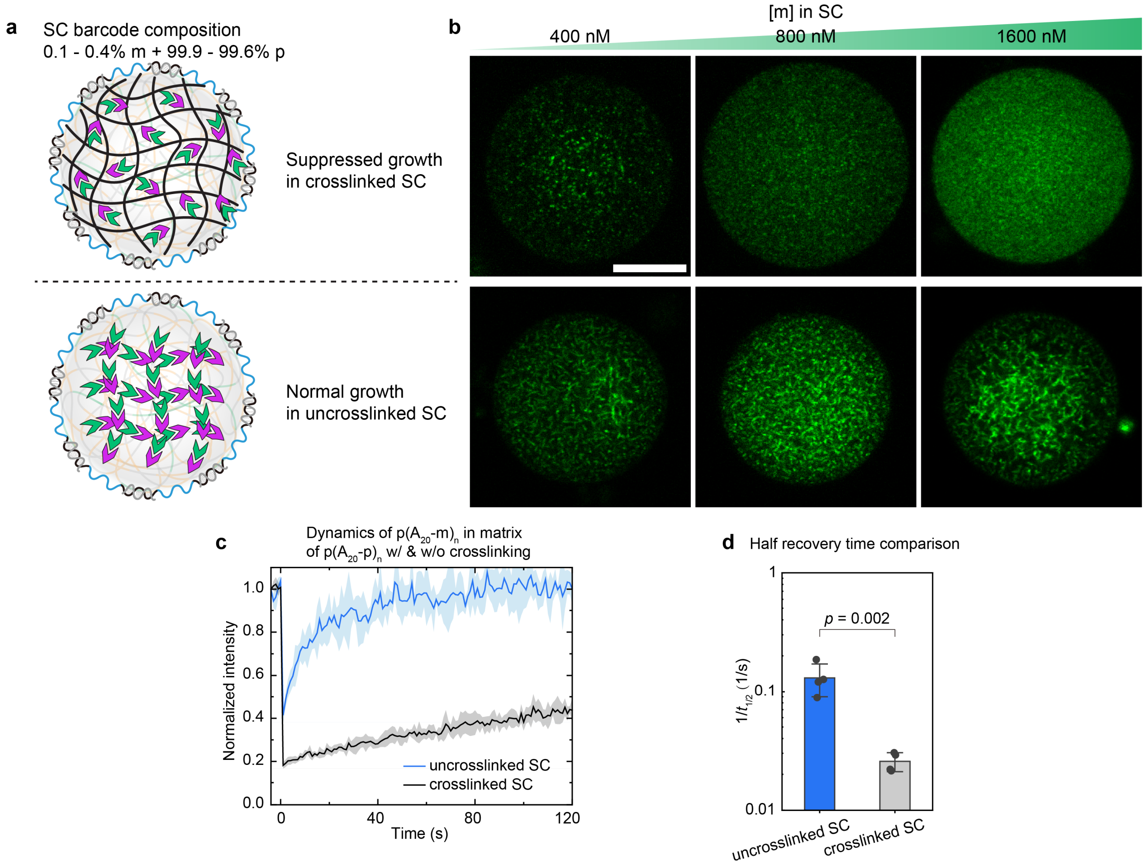

Supplementary Fig. 10: Crosslinking prevents cytoskeleton assembly.

a, Scheme for the DNT nucleation and growth behavior differences in crosslinked and uncrosslinked SCs. **b**, CLSM images depicting arrested and normal growth of DNTs in fully crosslinked $p(A_{20}\text{-}p)_n/p^*\text{-}A_5\text{-}p^*$ and uncrosslinked SCs (21 days) for different [m]. **c**, FRAP on the $p(A_{20}\text{-}m)_n$ (0.1%) chains labeled by Atto488-m* in the SCs with crosslinked and uncrosslinked $p(A_{20}\text{-}p)_n$ matrix (99.9%). The blue and black curves correspond to SCs in crosslinked and uncrosslinked states before the growth of artificial cytoskeletons (mean ± s.d., n = 4 FRAP experiments). **d**, Reciprocal of half recovery time ($1/t_{1/2}$) extracted from **c** (mean ± s.d., n = 4 FRAP experiments). The $1/t_{1/2}$ is a quantification for the internal dynamics of the SCs, which reflects their viscoelastic properties. For all CLSM images, the DNTs have two co-localized fluorescent labels (Atto488, Atto647). Only Atto488 (green) is shown for highest resolution. Experiment temperature = 20 °C. Scale bar, 10 μm (**b**).

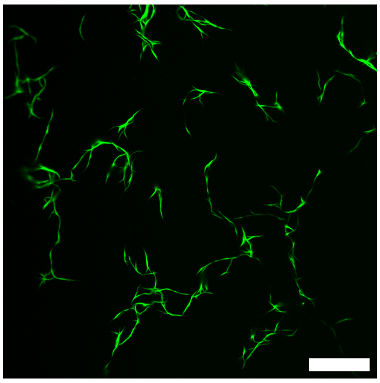

Supplementary Fig. 11: Representative CLSM image showing bundling of DNTs in a concentrated p(A_{20}-p)$_n$ solution.

We added 250 nM tile A and 250 nM tile B **without** binding strands to a concentrated p(A_{20}-p)$_n$ solution at 5 g/L in TE buffer with 15 mM $MgCl_2$, which nominally represents the SC interior. The DNA tiles do not bind to the p barcodes of p(A_{20}-p)$_n$. In this case, the DNA polymer p(A_{20}-p)$_n$ induces bundling of DNTs during their assembly. Such a structure is similar to the reported DNT bundling caused by crowding effects with simple crowders such as PEG or dextran. Experiment temperature = 20 °C. Scale bar, 20 µm.

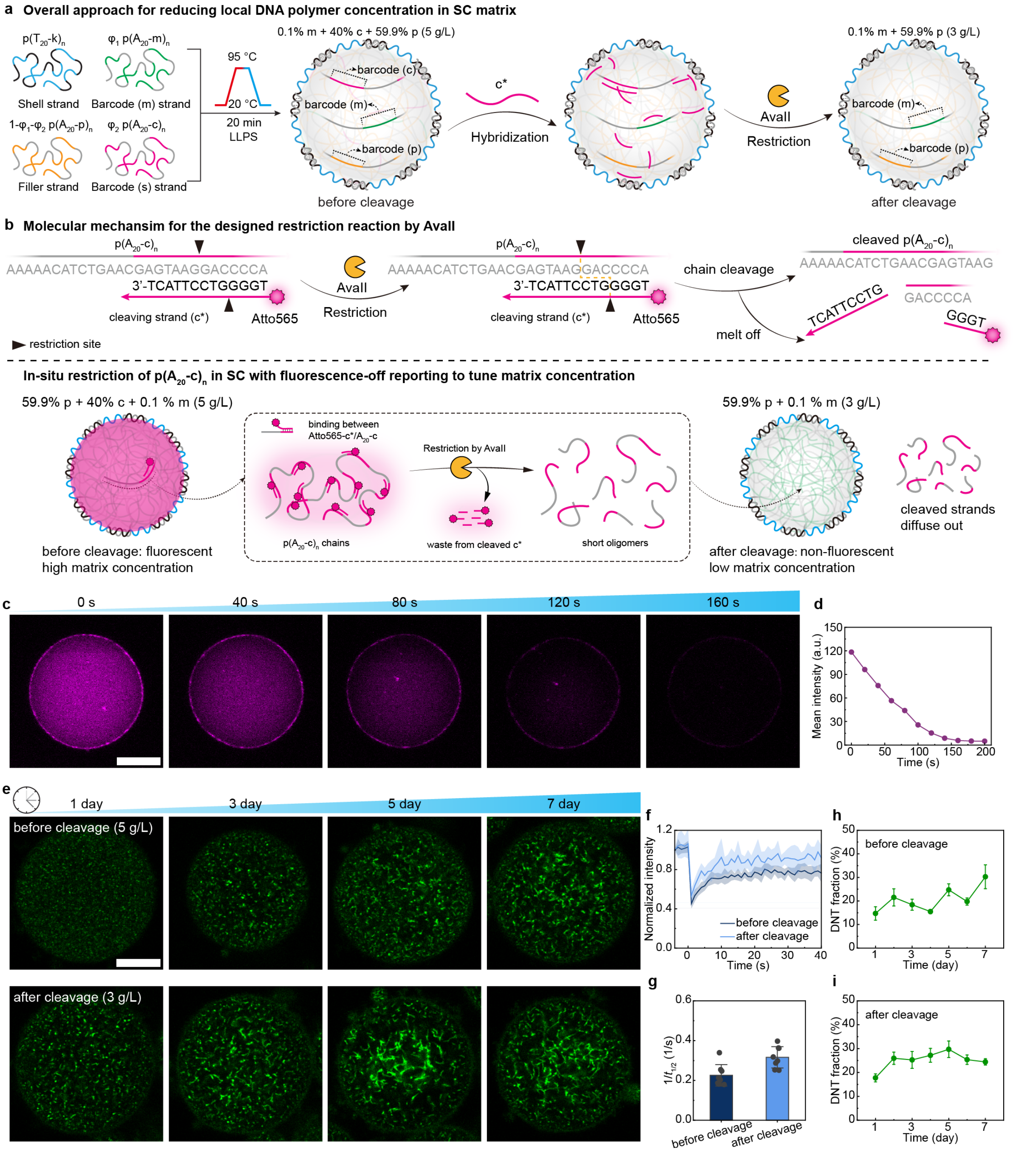

Supplementary Fig. 12: Reduction of DNA polymer concentration of SC matrix accelerates the DNT assembly inside SCs.

a, Scheme for the overall approach for reducing the local DNA polymer concentration inside the SC matrix. First, we prepared SCs based on co-phase-segregation of three pAs with different barcodes (0.1% m, 59.9% p, and 40% c). This is followed by hybridization to c* and enzymatic restriction by AvaII at c/c* dsDNA, leading to removal of the degradation products by diffusion **b**, Details of the enzymatic restriction by AvaII on $p(A_{20}\text{-}c)_n$ chains with help of a cleaving strand (Atto565-c*) inside the SC. Atto565-c* hybridizes to $p(A_{20}\text{-}c)_n$ and generates a recognition site for

the restriction enzyme AvaII. c* has 13 base pairs with p(A_{20}-c)$_n$ ($T_m \approx 69.7$ °C, analyzed with IDT Oligo Analyzer) and binds tightly at experimental temperature (20 °C). After cleavage, long polymer p(A_{20}-c)$_n$ will become short oligomers with 43 nt, which diffuse out of the SC with the cleaved Atto565-c*. The fluorophore at the 5' end of the c* serves as a reporter for monitoring the restriction as fluorescence vanishes from the SC into the surrounding environment. The final SC will only contain p(A_{20}-m)$_n$ and p(A_{20}-p)$_n$ with local concentration of DNA polymers at 3 g/L. **c**, Representative CLSM images showing the enzymatic restriction of a SC containing 59.9% p(A_{20}-p)$_n$, 40% p(A_{20}-c)$_n$, and 0.1% p(A_{20}-m)$_n$, loaded with Atto565-c* over time. **d**, Restriction kinetics indicated by decrease of fluorescence intensity within SC. **e**, Representative CLSM images showing the growth of DNA tiles inside the original SC with DNA matrix concentration at 5 g/L (top panel) and inside a SC after cleavage treatment with reduced DNA matrix concentration at 3 g/L (bottom panel) over time. **f**, FRAP on the p(A_{20}-m)$_n$ (0.1%) chains labeled by Atto488-m* in the SCs before and after cleavage treatment (mean ± s.d., n = 8 FRAP experiments). **g**, Reciprocal of half recovery time ($1/t_{1/2}$) extracted from **f** (mean ± s.d., n = 8 FRAP experiments). The $1/t_{1/2}$ is a quantification for the internal dynamics of the SCs, which reflects their viscoelastic properties. **h**, Growth of DNTs inside SCs before cleavage treatment, as quantified by the DNT fraction inside SCs as a function of time (mean ± s.d., n = 6 SCs measured). **i**, Growth of DNTs inside SCs after cleavage treatment, as quantified by the DNT fraction inside SCs as a function of time (mean ± s.d., n = 6 SCs measured). Experimental temperature = 20 °C. Scale bars, 10 μm (**c**, **e**).

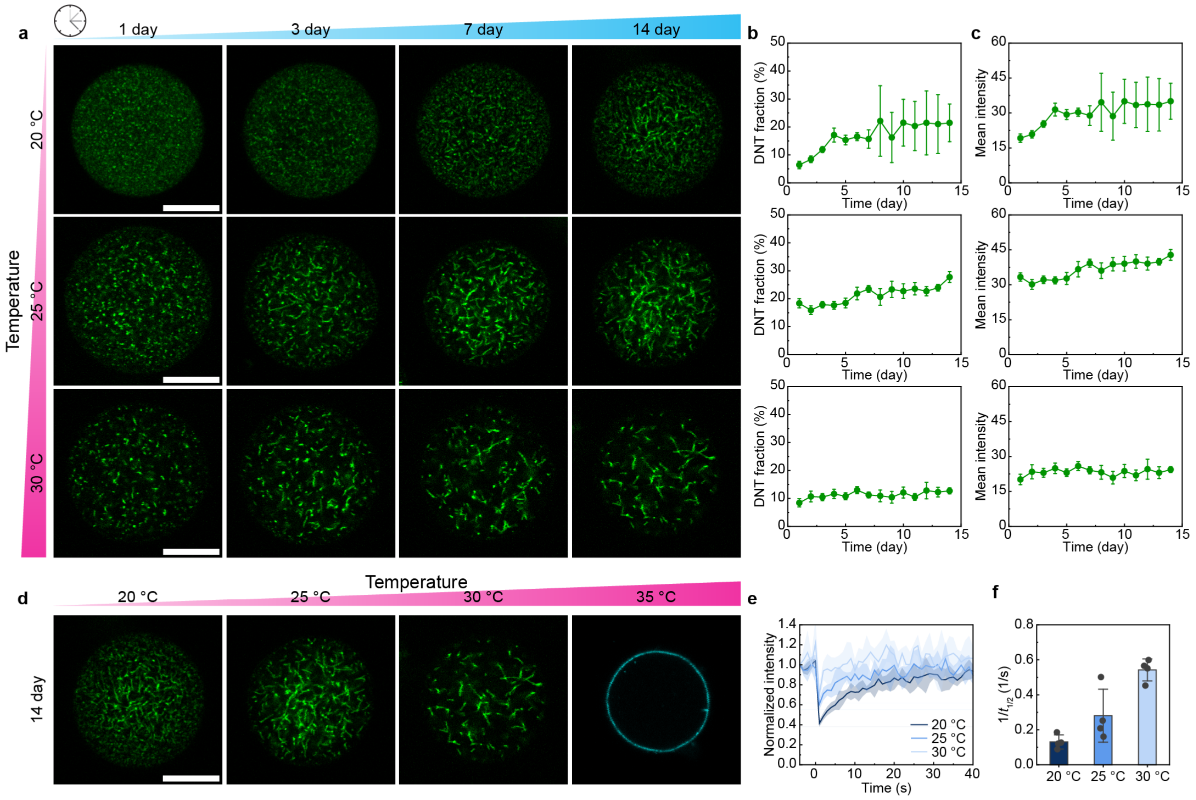

Supplementary Fig. 13: Formation of artificial cytoskeletons insides SCs at different temperatures.

a, Representative CLSM images showing the growth of DNTs inside SCs ([m] = 400 nM) at different temperatures over time. **b**, Growth of DNTs inside SCs at different temperatures, as quantified by the DNT fraction inside SCs as a function of time (mean ± s.d., n = 5 – 14 SCs measured at each day). **c**, Mean intensity of DNTs inside SCs at different temperatures as a function of time (mean ± s.d., n = 5 – 14 SCs measured at each day). **d**, Representative CLSM images comparing the growth of DNTs inside SCs after 14 days at different temperatures. **e**, FRAP on pristine $p(A_{20}\text{-}m)_n/p(A_{20}\text{-}p)_n$ SCs (0.1% m; 99.9% p) with simple labeling by Atto488-m* reveals accelerated recovery at higher temperature on account of increased reptation dynamics (mean ± s.d., n = 4 FRAP experiments). **f**, Reciprocal of half recovery time ($1/t_{1/2}$) extracted from **e** (mean ± s.d., n = 4 FRAP experiments). The $1/t_{1/2}$ is a quantification for the internal dynamics of the SCs, which reflects their viscoelastic properties. We note that the scaling law for concentration-viscosity relationship at entanglement regime for polymer solution does not hold due to the complexity of the studied polyelectrolyte solution complexed and interacting with bivalent counterions. Scale bars, 10 µm (**a**, **d**).

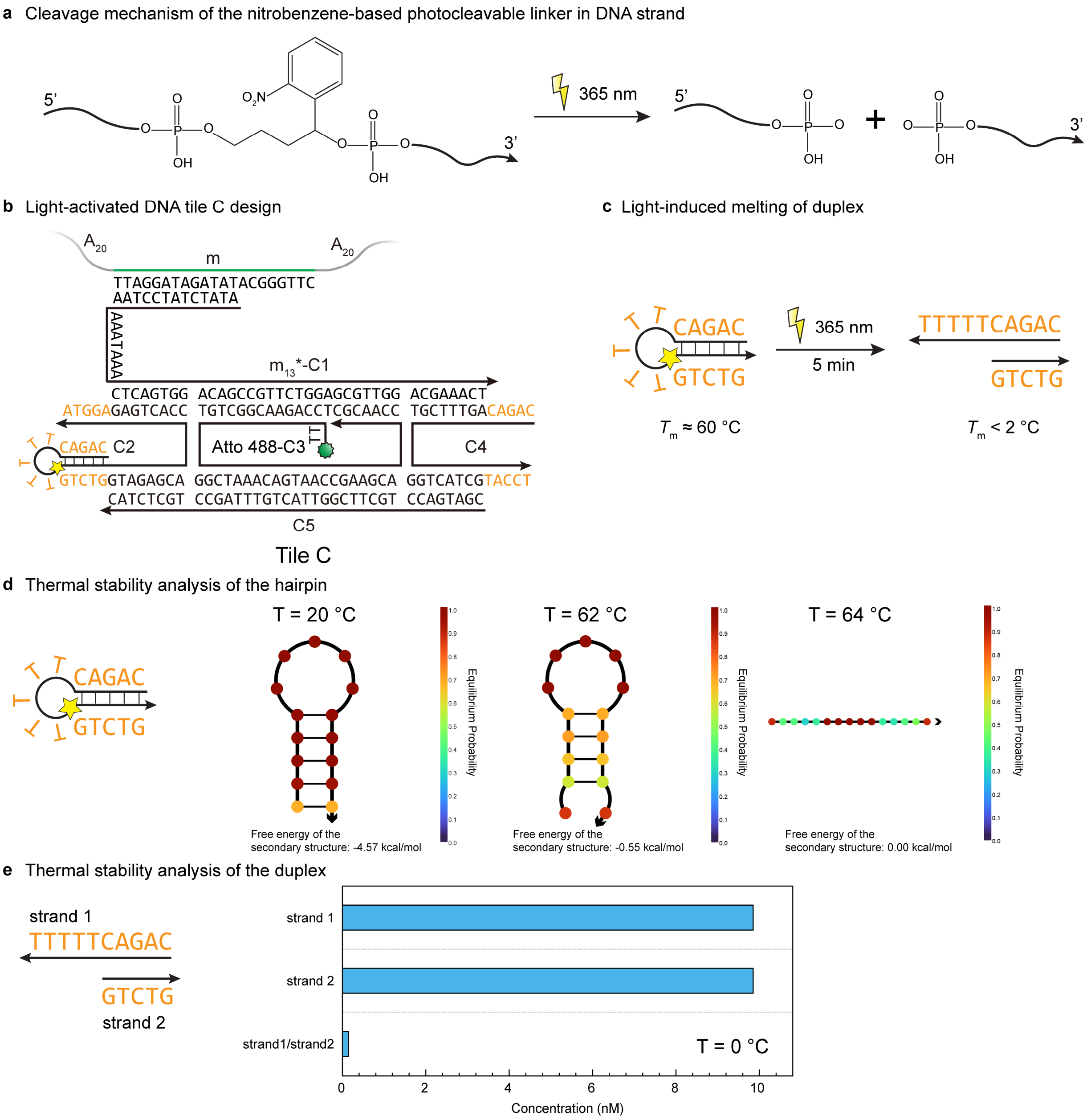

Supplementary Fig. 14: Light-activated DNT assembly using photocaged DNA tile C.
a, Cleavage mechanism of the photocleavable linker (nitrobenzene) used in this work. **b**, Design of the light-activated DNA tile, containing Atto488 and a dangling strand complementary to a part of the m barcode of p(A_{20}-m)$_n$. **c**, Schematic representation for the cleavage-induced melting of the duplex utilizing the significant difference in their melting temperatures (T_m). **d**, NUPACK analysis of thermal stability of the hairpin. **e**, NUPACK analysis of thermal stability of the duplex after the hairpin is cleaved. NUPACK condition for both analysis: 10 nM of strand, 50 mM Na^+, 15 mM Mg^{2+}.

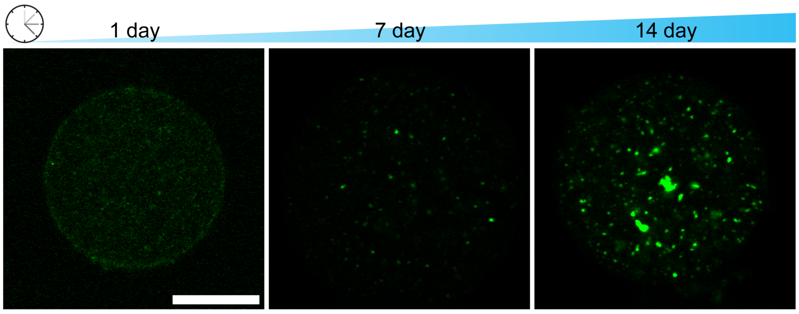

Supplementary Fig. 15: Control for light-activated artificial cytoskeleton inside SC shows absence of assembly.

Time-series CLSM images showing the absence of artificial cytoskeleton formation by tile C in a SC without UV illumination. Small aggregates appeared inside SC after 14 days, likely caused by ambient light pollution over long experimental time, which may have activated a small fraction of the initially inhibited tile C. Experimental temperature = 20 °C. Scale bar, 10 µm.

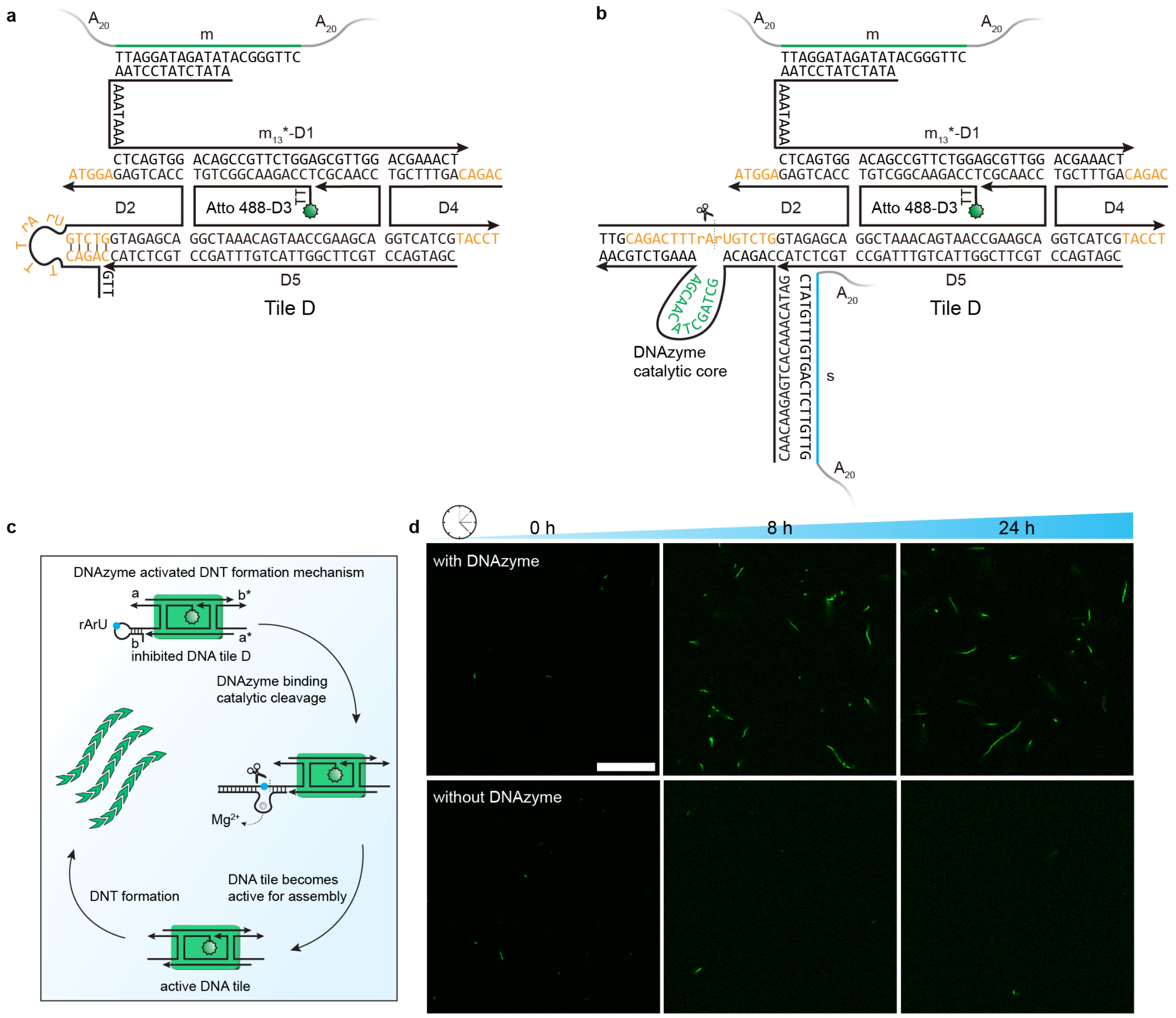

Supplementary Fig. 16: DNAzyme-catalyzed DNT assembly using DNA tile D in plain solution.

a, Design of the DNAzyme-catalyzed DNA tile D containing a dangling strand complementary to a part of the m barcode of p(A_{20}-m)$_n$. **b**, Design of DNAzyme and how the DNAzyme can bind to the tile D on its sticky end for catalytic cleavage of the RNA substrate (rArU unit). **c**, Schematic representation for the cleavage and assembly of tile D, activated by DNAzyme. **d**, Time-series CLSM images showing the formation of DNT in solution with DNAzyme over time. The control experiment without DNAzyme shows the absence of DNT formed. Experimental temperature = 20 °C. Scale bar 20 µm (**d**).

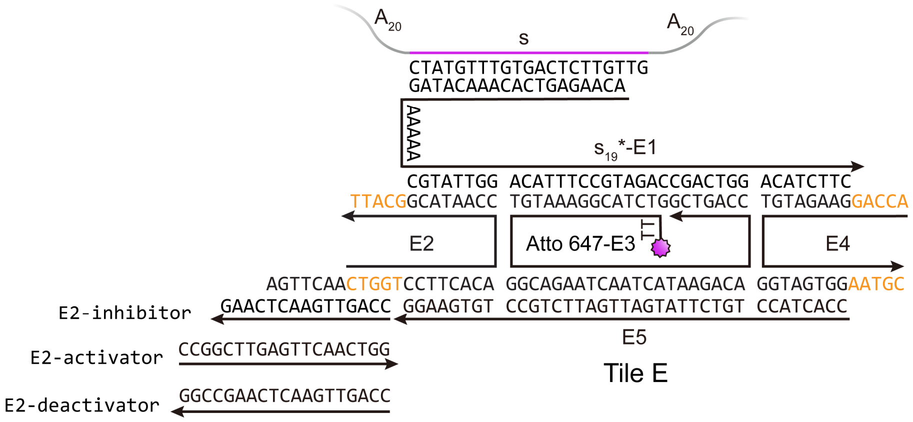

Supplementary Fig. 17: DSD-activated DNT assembly using DNA tile E.

Design of the DSD-activated DNA tile E containing a dangling strand complementary to a part of the s barcode of p(A_{20}-s)$_n$. This tile has a E2-inhibitor strand, which binds to one of the sticky ends and avoid autonomous formation of artificial cytoskeletons. The assembly of tiles can be triggered by adding E2-activator strand, which displaces the E2-inhibitor to set free the sticky end. The formed artificial cytoskeletons can be further deconstructed by adding E2-deactivator. E2-deactivator will displace the E2-activator so that the E2-inhibitor is available again in solution and will bind to the sticky ends to deconstruct the formed structures.

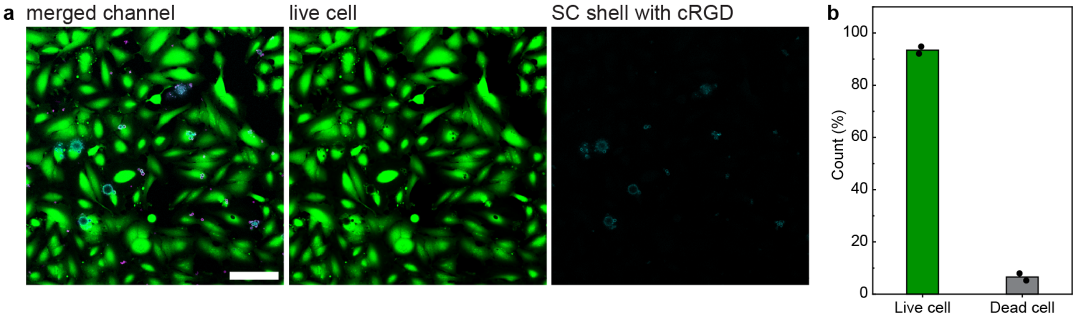

Supplementary Fig. 18: Cell viability after co-incubation with SCs.

a, Representative CLSM images in different channels for cell viability assay of co-incubated cells and SCs with cRGD at shell. The live cells are shown in green channel, the SC shells with cRGD are shown in cyan channel, and the dead cells and SCs are shown in magenta channel. The dead cells are labeled by SYTOX™ Deep Red Nucleic Acid Stain, which also labels the SC on the shell. **b**, Live and dead cell percentage for over 1000 cell counts. Co-incubation of cells and SCs with cRGD at shell was performed in a 96 well plate at 25 °C with 5 % CO_2 for 3 h. The co-incubation condition used here is the same as the experiments for SC-mammalian cell contact. (mean, n = 2 cell viability experiments for over 1000 cell counts in total). Scale bar, 100 µm (**a**).

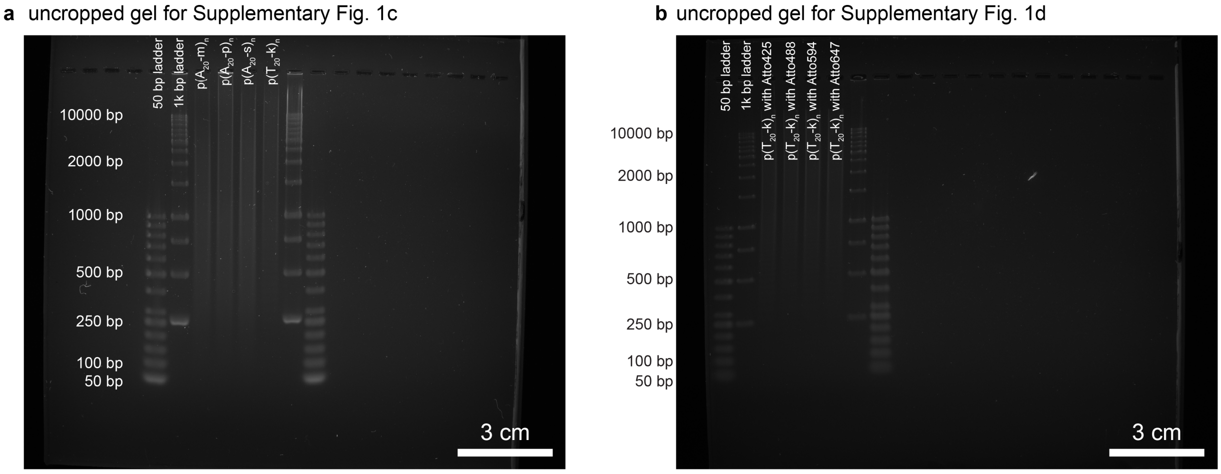

Supplementary Fig. 19. Uncropped gel scans.

a, Uncropped gel image for Supplementary Fig. 1c. **b**, Uncropped gel image for Supplementary Fig. 1d.

	SC composition	Starting materials	[m] or [s] in SC	[m] or [s] in solution (100× diluted)[1]	[DNA tile] in solution	Stoichiometry ([DNA tile] / [m] in solution)
Fig. 2	10% m<br>90% p	0.05 g/L $p(A_{20}\text{-}m)_n$<br>0.45 g/L $p(A_{20}\text{-}p)_n$	40000 nM [m]	40 nM [m]	10 nM tile A<br>10 nM tile B	0.5
	5% m<br>95% p	0.025 g/L $p(A_{20}\text{-}m)_n$<br>0.475 g/L $p(A_{20}\text{-}p)_n$	20000 nM [m]	20 nM [m]	10 nM tile A<br>10 nM tile B	1
	1% m<br>99% p	0.005 g/L $p(A_{20}\text{-}m)_n$<br>0.495 g/L $p(A_{20}\text{-}p)_n$	4000 nM [m]	4 nM [m]	10 nM tile A<br>10 nM tile B	5
	0.5% m<br>99.5% p	0.0025 g/L $p(A_{20}\text{-}m)_n$<br>0.4975 g/L $p(A_{20}\text{-}p)_n$	2000 nM [m]	2 nM [m]	10 nM tile A<br>10 nM tile B	10
	0.4% m<br>99.6% p	0.002 g/L $p(A_{20}\text{-}m)_n$<br>0.498 g/L $p(A_{20}\text{-}p)_n$	1600 nM [m]	1.6 nM [m]	10 nM tile A<br>10 nM tile B	12.5
	0.2% m<br>99.8% p	0.001 g/L $p(A_{20}\text{-}m)_n$<br>0.499 g/L $p(A_{20}\text{-}p)_n$	800 nM [m]	0.8 nM [m]	10 nM tile A<br>10 nM tile B	25
	0.1% m<br>99.9% p	0.0005 g/L $p(A_{20}\text{-}m)_n$<br>0.4995 g/L $p(A_{20}\text{-}p)_n$	400 nM [m]	0.4 nM [m]	10 nM tile A<br>10 nM tile B	50
	0.05% m<br>99.95% p	0.00025 g/L $p(A_{20}\text{-}m)_n$<br>0.49975 g/L $p(A_{20}\text{-}p)_n$	200 nM [m]	0.2 nM [m]	10 nM tile A<br>10 nM tile B	100
	0.025% m<br>99.975% p	0.000125 g/L $p(A_{20}\text{-}m)_n$<br>0.499875 g/L $p(A_{20}\text{-}p)_n$	100 nM [m]	0.1 nM [m]	10 nM tile A<br>10 nM tile B	200
Fig. 3	0.1% m<br>99.9% p	0.0005 g/L $p(A_{20}\text{-}m)_n$<br>0.4995 g/L $p(A_{20}\text{-}p)_n$	400 nM [m]	0.4 nM [m]	10 nM tile C	25
	0.1% m<br>10% s<br>89.9% p	0.0005 g/L $p(A_{20}\text{-}m)_n$<br>0.05 g/L $p(A_{20}\text{-}s)_n$<br>0.4495 g/L $p(A_{20}\text{-}p)_n$	400 nM [m]<br>40000 nM [s]	0.4 nM [m]<br>40 nM [s]	10 nM tile D	25
Fig. 4	0.1% m<br>0.1% s<br>99.9% p	0.0005 g/L $p(A_{20}\text{-}m)_n$<br>0.0005 g/L $p(A_{20}\text{-}s)_n$<br>0.4995 g/L $p(A_{20}\text{-}p)_n$	400 nM [m]<br>400 nM [s]	0.4 nM [m]<br>0.4 nM [s]	10 nM tile C<br>10 nM tile E	25
Fig. 5[2]	100% p	0.5 g/L $p(A_{20}\text{-}p)_n$	0 nM	0 nM	10 nM tile C<br>10 nM tile E	12.5
	0.1% m<br>99.9% p	0.0005 g/L $p(A_{20}\text{-}m)_n$<br>0.4995 g/L $p(A_{20}\text{-}p)_n$	400 nM [m]	0.4 nM [m]		
	0.1% s<br>99.9% p	0.0005 g/L $p(A_{20}\text{-}s)_n$<br>0.4995 g/L $p(A_{20}\text{-}p)_n$	400 nM [s]	0.4 nM [s]		
	0.1% m<br>0.1% s<br>99.9% p	0.0005 g/L $p(A_{20}\text{-}m)_n$<br>0.0005 g/L $p(A_{20}\text{-}s)_n$<br>0.4995 g/L $p(A_{20}\text{-}p)_n$	400 nM [m]<br>4000 nM [s]	0.4 nM [m]<br>0.4 nM [s]		
Fig. 6	0.15% m<br>99.85% p	0.00075 g/L $p(A_{20}\text{-}m)_n$<br>0.49925 g/L $p(A_{20}\text{-}p)_n$	600 nM [m]	0.6 nM [m]	10 nM tile A<br>10 nM tile B	33.33

[1] SCs were always used in 100× dilution with respect to the starting materials.

[2] For experiment shown in Fig. 4 in main text, 100× diluted SCs of each type are mixed together, resulting in 0.8 nM m barcode and 0.8 nM s barcode in the solution, with 10 nM tile C and 10 nM tile D added to grow two distinct artificial cytoskeletons.

Supplementary Table 1. Details about composition of SCs with varied internal barcode concentrations, barcode concentration in solution, DNA tile concentration in solution, and stoichiometry between DNA tile and barcode in individual experiments.

References

1 Chen, W. *et al.* Ballistic diffusion fronts in biomolecular condensates. *Nat. Nanotechnol.* (2025).

2 Colby, R. H. Structure and linear viscoelasticity of flexible polymer solutions: comparison of polyelectrolyte and neutral polymer solutions. *Rheol. Acta.* **49**, 425-442 (2009).

3 Samanta, A., Sabatino, V., Ward, T. R. & Walther, A. Functional and morphological adaptation in DNA protocells via signal processing prompted by artificial metalloenzymes. *Nat. Nanotechnol.* **15**, 914-921 (2020).

4 Merindol, R., Loescher, S., Samanta, A. & Walther, A. Pathway-controlled formation of mesostructured all-DNA colloids and superstructures. *Nat. Nanotechnol.* **13**, 730-738 (2018).
